# Controlling Cell Fate Specification System by Key Genes Determined from Network Structure

**DOI:** 10.1016/j.isci.2018.05.004

**Published:** 2018-06-07

**Authors:** Kenji Kobayashi, Kazuki Maeda, Miki Tokuoka, Atsushi Mochizuki, Yutaka Satou

**Affiliations:** 1 Department of Zoology, Graduate School of Science, Kyoto University, Sakyo, Kyoto 606-8502, Japan; 2 CREST, Japan Science and Technology Agency, 4-1-8 Honcho, Kawaguchi, Saitama 332-0012, Japan; 3 Department of Mathematical Sciences, School of Science and Technology, Kwansei Gakuin University, 2-1 Gakuen, Sanda, Hyogo 669-1337, Japan; 4 Theoretical Biology Laboratory, RIKEN, Wako, Saitama 351-0198, Japan; 5 Institute for Frontier Life and Medical Sciences, Kyoto University, Sakyo, Kyoto 606-8507, Japan

**Keywords:** Bioinformatics, Mathematical Biosciences, Complex System Biology, Experimental Models in Systems Biology

## Abstract

Network structures describing regulation between biomolecules have been determined in many biological systems. Dynamics of molecular activities based on such networks are considered to be the origin of many biological functions. Recently, it has been proved mathematically that key nodes for controlling dynamics in networks are identified from network structure alone. Here, we applied this theory to a gene regulatory network for the cell fate specification of seven tissues in the ascidian embryo and found that this network, which consisted of 92 factors, had five key molecules. By controlling the activities of these key molecules, the specific gene expression of six of seven tissues observed in the embryo was successfully reproduced. Since this method is applicable to all nonlinear dynamic systems, we propose this method as a tool for controlling gene regulatory networks and reprogramming cell fates.

## Highlights

• The linkage logic theory identifies key nodes in regulatory networks from structures
• It identified five key molecules in the gene regulatory network (GRN) of an ascidian embryo
• The network dynamics was controlled by manipulating activities of these key molecules

## Graphical Abstract

**Figure:**
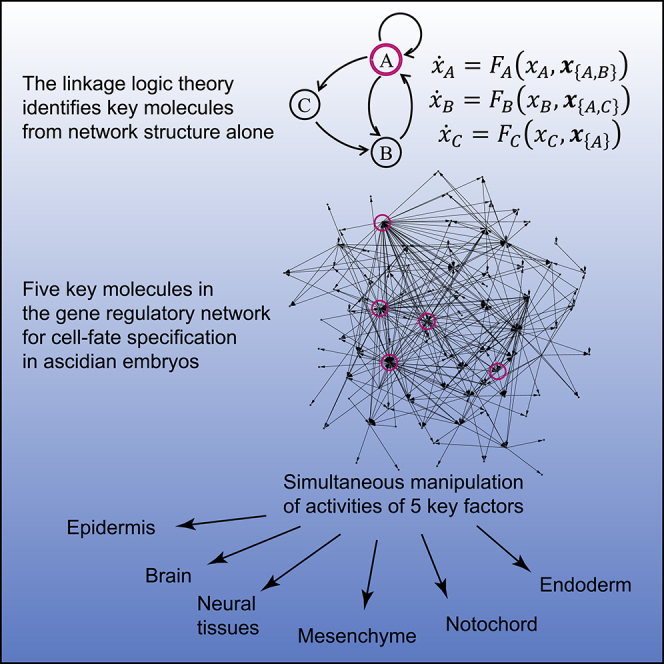

## Introduction

Network systems produce dynamics of molecular activity in organisms, and such dynamics are thought to be the origin of biological functions (Alon, 2007, Oda et al., 2005, Peter and Davidson, 2016). A variety of cell types originate in the diversity of steady states of gene expression. We recently developed a new theoretical framework (linkage logic theory) (Fiedler et al., 2013, Mochizuki, 2008, Mochizuki et al., 2013), with which key nodes for controlling nonlinear dynamics are identified only from network structure without assuming quantitative details, such as functional forms, parameters, or initial states. According to this theory, the dynamics of a system is controllable to converge on any solution by controlling a subset of nodes called a feedback vertex set (FVS). Therefore, if the dynamics of a GRN has multiple steady states, we should be able to reproduce them and control the dynamics of the system by manipulating the activities of FVS molecules alone.

In the present study, we applied the linkage logic theory to a GRN that specifies cell fates in embryos of the ascidian *Ciona intestinalis* (type A; also called *Ciona robusta*). The network structure for the specification of cell fate has been determined by a genome-wide gene knockdown assay for regulatory genes that are expressed during embryogenesis (Imai et al., 2006) and was recently updated using data that had been accumulated after the initial construction (Satou and Imai, 2015). Hence, if the fate decision is based on the steady states of this network, cell-type-specific gene expression patterns should be reproduced by manipulating the activities of FVS in the network. Here, we show that the minimum FVSs of this network contain only five factors and that the dynamics of the GRN is indeed controllable by these five FVS factors.

## Results

### Controlling Nonlinear Dynamics of Networks

First, we show the linkage logic theory is applicable to GRNs. A GRN is represented by a directed graph $\Gamma =\left(V,E\right)$, consisting of a node set *V* and an edge set *E*, where nodes represent genes and edges represent regulatory linkages. The dynamics of gene activities is modeled by a system of ordinary differential equations. We assume that gene activities, measured in terms of the concentrations of mRNAs or proteins, decay in the absence of supply or synthesis. Suppose that the dynamics of activity *x*_*n*_ of gene n∈V is written in the form:

(Equation 1) $$\begin{array}{c} {\dot{x}}_{n}=F_{n}\left(x\right)\\ \;=F_{n}\left(x_{n},\;x_{I_{n}}\right) \end{array}$$

with the “decay condition”:

(Equation 2) $$\partial_{1}F_{n}\left(x_{n},x_{I_{n}}\right)<\;0.$$

**Figure 1:**
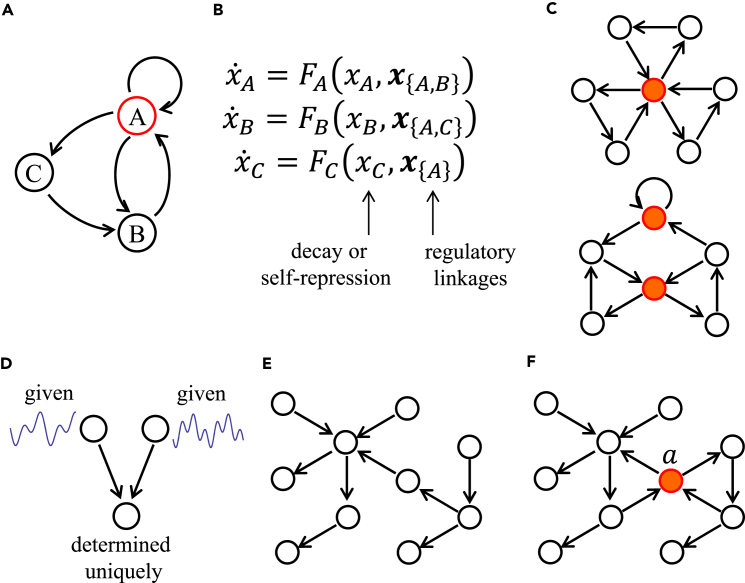
Controlling Network Dynamics by FVS (A) A GRN containing three nodes and three cycles (*A* → *A*, *A* → *B*→ *A*, *A* → *C* → *B* → *A*). Regulatory interaction is positive for the self-loop in *A* and either positive or negative for the others. The minimum FVS includes node *A*. (B) An ODE system corresponding to the network in (A). The second argument sets in *F*_*n*_s specify regulatory linkages. (C) Two GRNs containing minimum FVSs marked in red. (D) The dynamics of regulating nodes determines the dynamics of the regulated node uniquely. (E) A network without cycles has an empty FVS. (F) A network with two cycles has an FVS including node *a* only.

The set $I_{n}\subseteq V$ is the input set of *n*, a subset of molecules that regulate molecule *n*, that is, $I_{n}=\left\{i|\left(i\to n\right)\in E\right\}$. The notation $\partial_{1}$ implies the first partial derivative with respect to the first argument. The set *I*_*n*_ includes *n* $\left(n\in I_{n}\right)$, that is, it is a self-regulatory loop, if $\partial F_{n}/\partial x_{n}$ is “not always negative.” Note that even if $\partial F_{n}/\partial x_{n}$ is not negative, we can make the system satisfy the decay condition (2) by adding a positive term indicating a self-regulatory loop. The sets of *I*_*n*_ (∀n∈V) directly represent the graphical structure of the regulatory network. An example of a hypothetical network consisting of three genes is shown in Figures 1A and 1B.

Under formulations (1) and (2), we proved that sets of key nodes for dynamics are determined from the topology of the network (Fiedler et al., 2013, Mochizuki, 2008, Mochizuki et al., 2013) as FVSs. In graph theory, an FVS is defined as a subset of vertices in a directed graph whose removal leaves a graph without directed cycles (Akutsu et al., 1998). In the above hypothetical network, gene *A* constitutes the minimum FVS (Figure 1A). Two additional examples are shown in Figure 1C. Here, we give an intuitive explanation of our theory using illustrative examples (see Methods for details). (1) In a simple regulatory system including two regulator nodes and a regulated node (Figure 1D), if the dynamics of the regulator nodes is given, the dynamics of the regulated node is determined uniquely; that is, for any initial state, the dynamics of the node converges to a single trajectory for a long time. (2) In a GRN without a cycle (Figure 1E), the dynamics of “top” nodes, which receive no regulatory input, converges on the unique equilibrium. By determining the dynamics of each node downward through the network, the dynamics of a system without a cycle should converge on a unique equilibrium, which is globally stable. (3) Then, consider a GRN including two cycles as shown in Figure 1F, the corresponding dynamics of which can have multiple solutions. Say it has two steady states *S*_1_ and *S*_2_. Node *a* is a single element of FVS because the graph without node *a* includes no cycle. Suppose we fix the activity level of node *a* to be equal to the value at steady state *S*_1_ by experimental manipulation. Then, the remaining nodes constitute a graph without a cycle, and the dynamics of these nodes spontaneously converges on the unique steady state, which must be the same as the steady state *S*_1_ in the original system. If instead we fix the activity level of the node equal to the value of steady state *S*_2_, then the dynamics of other genes converges on the unique equilibrium that is equal to steady state *S*_2_. Note that the dynamics of the top nodes, which receive no regulatory input, converges on the unique equilibrium. Hence, by experimentally manipulating a single node, we can make the system converge on steady state *S*_1_ or steady state *S*_2_ as required. When FVSs include multiple nodes, all of them must be fixed simultaneously to control the dynamics to converge on desired steady states.

The controllability by FVS has a broader meaning than switching between solutions that can be observed in natural conditions. For any fixed value of nodes in an FVS, the dynamics of other nodes, which are not included in the FVS, converges on a unique steady state, even if the given value is not chosen from known natural steady states. This implies that an exhaustive search of steady states is possible under an assumption of discreteness. If we assume that all possible steady states in a given system have binary values, that is, 0 or 1, on an FVS, we can examine all combinations of manipulations of nodes in the FVS. The obtained set of the states should include all the natural steady states of the system.

### FVSs of the Gene Regulatory Network for Fate Specification in Ascidian Embryos

**Figure 2:**
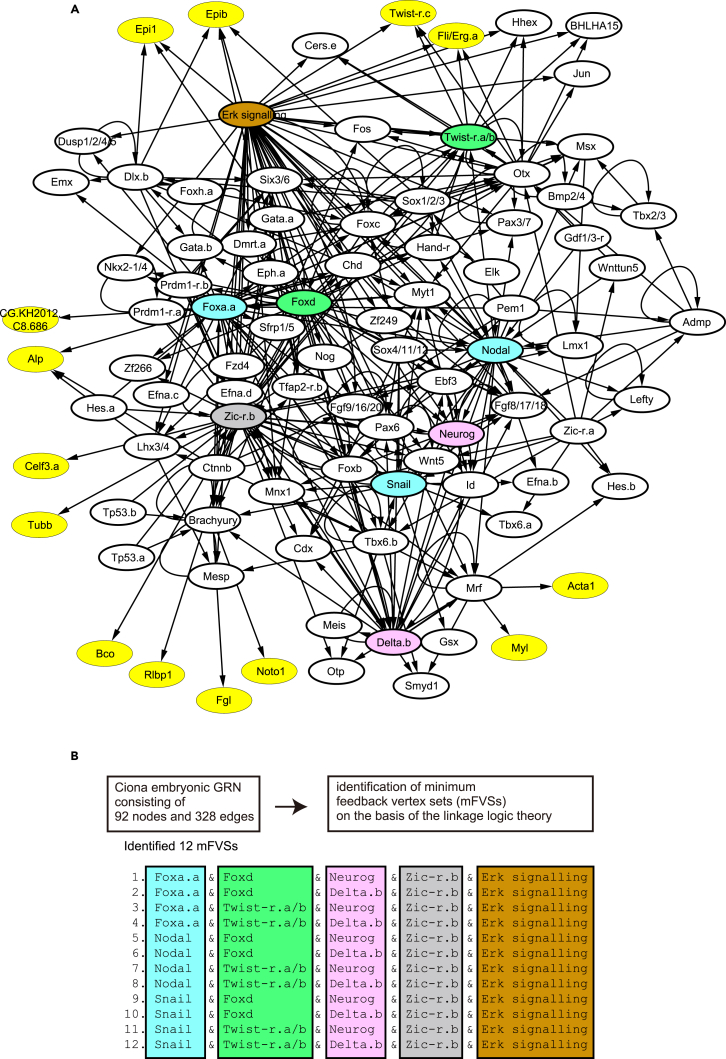
Gene Regulatory Network for Cell Specification in *Ciona* (A) The GRN consists of 92 factors (nodes) and 328 regulatory interactions (edges). The network possessing minimum FVS consists of five nodes. The 12 choices of the node sets are given by choosing a single node from each of five node sets colored light blue, green, pink, gray, and orange. Nodes filled in yellow are the marker genes, for which we performed observations of activities. See also Figure S1 and S2. (B) List of nodes in the 12 minimum FVSs.

We tested the FVS controllability using the GRN to specify cell fates in an ascidian embryo (Imai et al., 2006, Satou and Imai, 2015). Before the late gastrula stage of *Ciona* embryos, the cell fate of each blastomere is restricted to one of seven tissues, epidermis, brain, nerve cord, endoderm, notochord, mesenchyme, or muscle, which exhibit specific gene expression patterns in their descendants at later stages. Zygotic expression starts between the 8- and 16-cell stages, and the dynamics of gene expression until the late gastrula stage specifies the developmental fates of the above-mentioned seven tissues. The GRN responsible for specification of these cell fates includes 92 genes and 328 regulatory linkages (Figures 2A and S1). From an analysis of the structure of the network (see Methods), we identified 12 minimum FVSs, each of which contained five genes (color outlined in Figure 2). The minimum FVSs are {*Foxa.a*|*Nodal*|*Snail*, *Foxd*|*Twist-r.a/b*, *Neurog*|*Delta.b*, *Zic-r.b*, Erk signaling}, where “|” indicates an alternative choice (3 × 2 × 2 × 1 × 1 = 12 sets; see Figure 2B). The existence of FVSs indicates that the GRN potentially possesses multiple steady states. If the activity of the FVS factors is assumed to be binary, that is, active or inactive, all steady states will be obtained by up- and down-regulation of the activities of molecules in an FVS, as discussed earlier.

**Figure 3:**
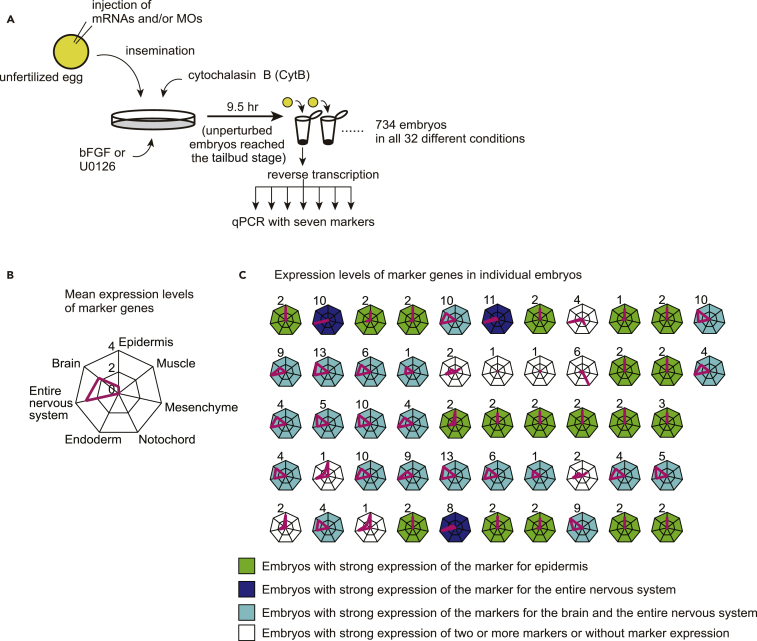
The Experimental System Using Syncytium Embryos (A) Depiction of the experimental design for testing the prediction of the linkage logic theory. (B and C) Syncytium embryos treated with CytB expressed epidermal and neural markers. Expression levels of marker genes are shown relative to the corresponding values in normal 9.5-hr (tailbud-stage) embryos. (B) Mean values and (C) values of individual embryos are shown. In (B), in these experimental embryos, two markers for the brain and the entire nervous system were predominantly expressed, and an epidermal marker was also expressed. Other markers were rarely expressed. The axes in (C) show expression of the same tissue marker genes as in (B). Embryos that predominantly expressed the epidermal marker, a set of the pan-neural and brain markers, and pan-neural marker are shown in green, light blue, and blue, respectively.

In multicellular embryos, GRNs encoded in individual cells are mutually connected through intercellular interactions and function as subnetworks to constitute a larger GRN. In addition, such interactions are affected by three-dimensional structures unique to various stages of embryos. To avoid such possible effects, we developed an experimental system of single-cell development by treating fertilized eggs with cytochalasin B (CytB) (Figure 3A). Although cells in CytB-treated embryos never divide, nuclear divisions continue, and specification dynamics is considered to proceed (Hudson et al., 2003, Hudson and Yasuo, 2006, Jeffery et al., 2008, Kodama et al., 2016, Meedel et al., 2007, Oda-Ishii and Di Gregorio, 2007, Satoh, 1979, Shi and Levine, 2008, Tokuoka et al., 2004, Whittaker, 1973, Yasuo and Hudson, 2007). We did not exclude signaling molecules from our analysis, because signaling molecules could work in an autocrine manner. To identify cell fates by reverse transcription-quantitative PCR (RT-qPCR) and *in situ* hybridization, we chose the following genes as markers: *Epi1* and *Epib* for the epidermis, *Bco* and *Rlbp1* for the brain, *Celf3.a* and *Tubb* for the entire neural system, *Alp* and *CG.KH2012.C8.686* for the endoderm, *Noto1* and *Fgl* for the notochord, *Fli/Erg.a* and *Twist-r.c* for the mesenchyme, and *Myl* and *Acta1* for the muscle (Chiba et al., 1998, Hotta et al., 1999, Imai et al., 2000, Imai et al., 2003, Imai et al., 2004, Kusakabe et al., 2002, Satou et al., 2001, Takahashi et al., 1999, Ueki et al., 1994, Yagi and Makabe, 2001). We confirmed that these marker genes were indeed regulated by *Dlx.b*, *Zic-r.b*, *Foxa.a*, *Brachyury*, *Twist-r.a/b*, or *Mrf*, which were included in the above-mentioned GRN (Figure S2).

To confirm that specification dynamics proceeds in CytB-treated embryos and to examine whether the cell fate specification in CytB-treated embryos is deterministic, we used RT-qPCR and measured the expression of seven marker genes, *Epi1*, *Bco*, *Celf3.a*, *Alp*, *Noto1*, *Fli/Erg.a*, and *Myl*, for seven different tissues in 52 embryos at 9.5 hr after fertilization, which corresponded to the tailbud stage in normal embryos (Figures 3B and 3C; Table 1). Expression levels of marker genes were measured relative to the corresponding values in normal 9.5 hr embryos. Among the 52 embryos, 19, 21, and 3 strongly expressed *Epi1*, a set of *Bco* and *Celf3.a*, and *Celf3.a*, respectively. Namely, the marker gene expression patterns in these embryos resembled those in epidermal, brain, and nerve cord cells. Seven embryos expressed marker genes for multiple tissues, and the remaining two embryos rarely expressed marker genes. Under the manipulation of CytB treatment, cell specification became nondeterministic, and the resultant diversity of marker gene expression was smaller than in normal embryos. In contrast, the observation that marker genes were expressed at 9.5 hr after fertilization suggested that the dynamics of the GRN for cell fate specification proceeded in CytB-treated embryos.

**Table 1:** Mean Expression Levels of Marker Genes in Nine Representative Conditions

		Experimental Condition^a^								
		Unperturbed	adnze	adnZe	adNze	Adnze	aDnze	adnZE	adnz	Z
Expression^b^	*Epi1* (epidermis)	0.63	0.91	0.00	0.00	0.00	0.00	0.00	0.50	0.00
	*Bco* (brain)	2.30	0.08	2.20	0.06	0.15	0.14	0.43	0.18	2.16
	*Celf3.a* (pan-neural)	3.03	0.15	3.61	9.59	0.19	0.17	0.18	0.34	2.60
	*Alp* (endoderm)	0.01	0.02	0.03	0.13	1.94	0.83	0.01	0.04	0.03
	*Noto1* (notochord)	0.15	0.01	0.01	0.00	0.31	2.07	0.00	0.01	0.01
	*Fli/Erg.a* (mesenchyme)	0.00	0.00	0.00	0.00	0.01	0.00	6.21	0.00	4.76
	*Myl* (muscle)	0.04	0.00	0.00	0.00	0.00	0.01	0.01	0.00	0.08

a Each of the experimental conditions is represented by a five-letter code in which up- and down-regulation of *Foxa.a*, *Foxd*, *Neurog*, *Zic-r.b*, and Erk signaling are represented by A/a, D/d, N/n, Z/z, and E/e, respectively. See also Table S1.

b Expression levels of marker genes are shown relative to the corresponding values in normal 9.5-hr (tailbud-stage) embryos.

### Dynamics of the Network for Cell Fate Specification Was Controllable by Manipulating the Activities of the FVS Factors

Among the 12 minimum FVSs, we chose an FVS consisting of *Foxa.a*, *Foxd*, *Neurog*, *Zic-r.b*, and Erk signaling because we have morpholino antisense oligonucleotides that are effective for the knockdown of *Foxa.a*, *Foxd*, *Neurog*, and *Zic-r.b* (Hudson et al., 2016, Imai et al., 2006). In addition, we used synthetic mRNAs for up-regulation of the activities of these genes. For the up- and down-regulation of Erk signaling, we added a recombinant FGF protein and an MEK inhibitor to seawater. Using these experimental tools, we performed exhaustive manipulation in a binary manner (i.e., up- or down-regulation of these FVS factors; 2^5^ = 32 combinations) to identify all possible steady states that the system reaches.

**Figure 4:**
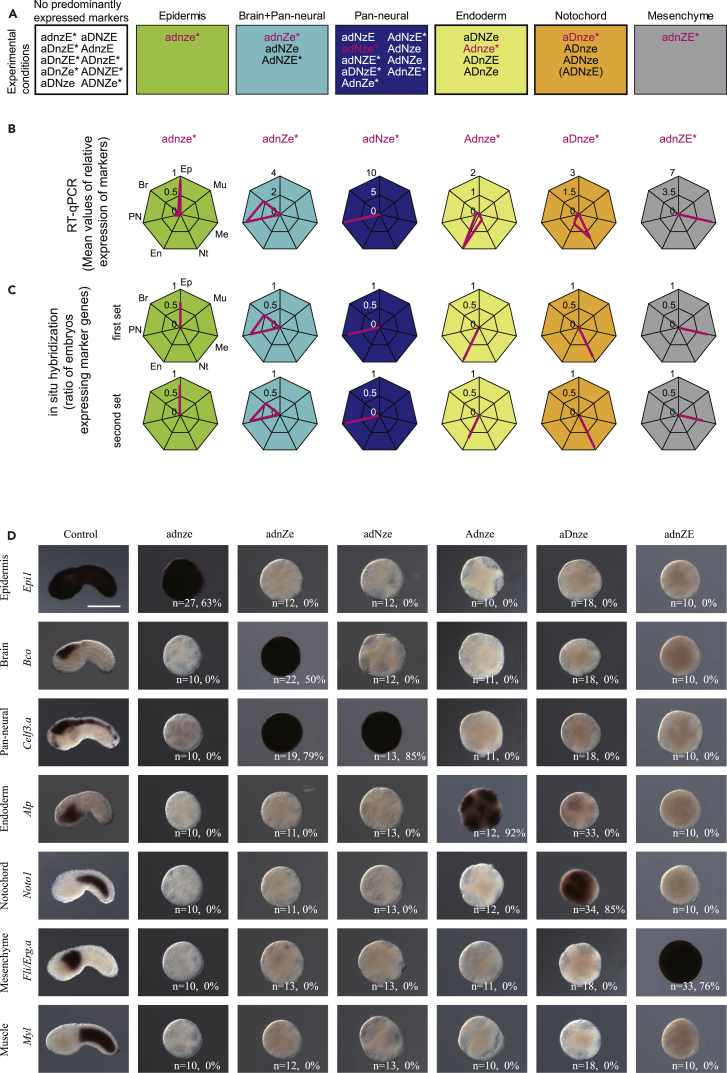
Expression of Marker Genes by Manipulation of the Activities of the FVS Factors (A–C) Marker expression in experimental embryos. Conditions with asterisks were examined in multiple batches. Each of the experimental conditions is represented by a five-letter code in which up- and down-regulation of *Foxa.a*, *Foxd*, *Neurog*, *Zic-r.b*, and Erk signaling are represented by A/a, D/d, N/n, Z/z, and E/e, respectively. (A) Markers predominantly expressed in 32 experimental conditions. See also Figures S3–S7. (B and C) The RT-qPCR results for six representative conditions shown in magenta are presented in (B) and were further examined by *in situ* hybridization as shown in (C). The axes of the first graph are labeled: Ep, epidermal marker; Br, brain marker; PN, pan-neural marker; En, endodermal marker; Nt, notochord marker; Me, mesenchyme marker; Mu, muscle marker. This configuration is applied to the other graphs. In (C), the results for the original set (upper; photographs are shown in D) and an additional set of markers (lower; photographs are shown in Figure S8) are shown. (D) *In situ* hybridization of the first set of marker genes shown in (C). Gene names are shown on the left. The numbers of embryos we examined and the percentage of embryos that expressed the markers are shown in each panel. Scale bar, 100 μm. See also Figure S8.

We examined a total of 734 embryos by RT-qPCR for seven marker genes including at least 12 embryos of a single batch for each of the 32 conditions (Figures S3–S6; Table S1). The expression of marker genes was deterministic in most cases under the manipulation of FVS factors. Namely, the embryos under the same manipulating condition exhibit almost the same pattern of expression of the marker genes. We applied sign tests to examine whether a single tissue marker or a set of *Bco* and *Celf3.a* was predominantly expressed in each of the 32 conditions (Figure S7). The expression of marker genes is summarized in Figure 4. Each of the experimental conditions is represented by a five-letter code in which up- and down-regulation of *Foxa.a*, *Foxd*, *Neurog*, *Zic-r.b*, and Erk signaling are represented by A/a, D/d, N/n, Z/z, and E/e, respectively; for example, embryos exhibiting up-regulation of *Foxa.a* and down-regulation of the other factors are referred to as Adnze. As shown in Figure 4, in 22 conditions, *Epi1*, a set of *Bco* and *Celf3.a*, *Celf3.a*, *Alp*, *Noto1*, or *Fli/Erg.a* was expressed (Figure 4A). Such gene expression patterns were similar to those in cells of the epidermis, brain, nerve cord, endoderm, notochord, and mesenchyme, respectively. In contrast, we rarely observed simultaneous expression of markers for multiple tissues in a single embryo. Figure 4B shows the average relative gene expression in six representative conditions (see also Table 1). In these six conditions, we also performed *in situ* hybridization with the same set of markers and an additional set of markers, *Epib*, *Rlbp1*, *Tubb*, *CG.KH2012.C8.686*, *Fgl*, and *Twist-r.c* (Figures 4C, 4D, and S8). The results were consistent with those of the aforementioned RT-qPCR. The observation that gene expression patterns did not basically differ among individual embryos in each condition indicated that manipulation of the activities of the FVS factors was deterministic.

### Dynamics of the Network for Cell Fate Specification Was Not Controllable by Manipulating the Activities of a Subset of the FVS Factors

**Figure 5:**
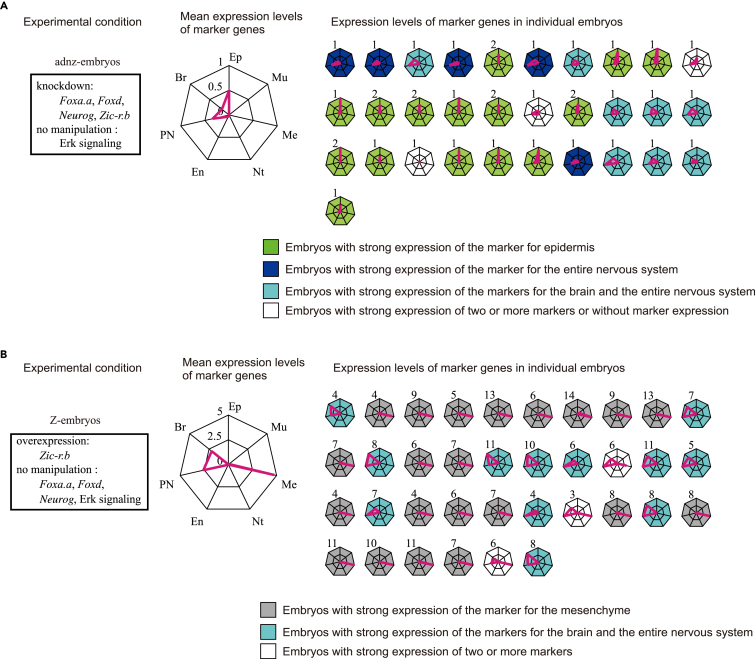
Dynamics of the Network for Cell Fate Specification Is Uncontrollable by Manipulating the Activities of a Subset of the FVS Factors (A and B) Marker gene expression determined by RT-qPCR (A) in adnz-embryos and (B) in Z-embryos. Mean values (left large graphs) and all values for individual embryos (right small graphs) are shown. The axes of the first graph are labeled: Ep, epidermal marker; Br, brain marker; PN, pan-neural marker; En, endodermal marker; Nt, notochord marker; Me, mesenchyme marker; Mu, muscle marker. This configuration is applied to the other graphs. Different colors in the small graphs indicate that different tissue markers are predominantly expressed.

In contrast to the findings described earlier, the manipulation of activities of a subset of the FVS factors (*Foxa.a*, *Foxd*, *Neurog* and *Zic-r.b*, but not Erk signaling) did not drive the GRN dynamics deterministically into a single steady state. Namely, gene expression patterns differed among the individual embryos that expressed *Epi1*, a set of *Bco*/*Celf3.a*, or *Celf3.a* (Figure 5A; Table 1), as gene expression patterns differed in embryos without manipulation of the activities of the FVS factors (Figures 3B and 3C). Similarly, overexpression of *Zic-r.b* alone did not determine cell fate uniquely, either; namely, such embryos expressed *Fli/Erg.a*, a set of *Bco*/*Celf3.a*, or both (Figure 5B; Table 1). These observations were consistent with a proposition of the linkage logic theory, namely, that manipulation of the whole FVS is necessary to fully control network dynamics.

### Gene Expression Profiles of Induced Notochord and Mesenchyme

**Figure 6:**
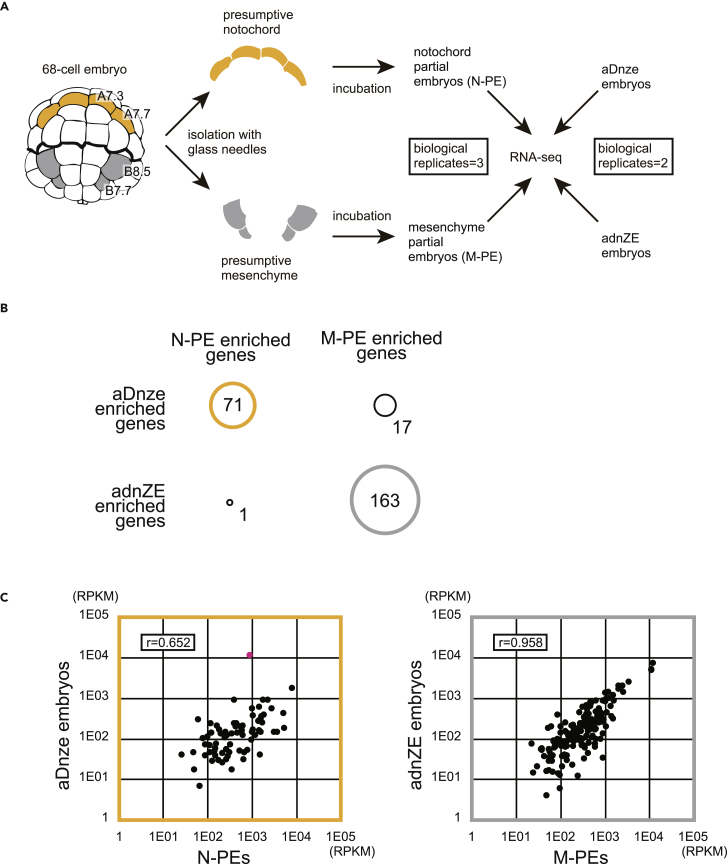
Analysis of Expression Profiles for aDnze and adnZE Embryos (A) The experimental design for RNA sequencing of partial embryos of notochord and mesenchyme (N-PE and M-PE) and of aDnze and adnZE embryos. (B) Comparisons between gene fractions enriched in N-PE and M-PE and those enriched in aDnze and adnZE embryos. (C) Scatterplots showing expression levels of the 71 genes found commonly between gene fractions enriched in N-PE and aDnze embryos and those of the 163 genes found commonly between gene fractions enriched in M-PE and adnZE embryos.

Finally, we compared the genome-wide expression profiles of embryos in two conditions (aDnze and adnZE), in which notochord and mesenchyme markers were predominantly expressed, with those of notochord and mesenchyme cells (Figure 6). For this purpose, we isolated two pairs of presumptive notochord cells and two pairs of presumptive mesenchyme cells because these isolated blastomeres differentiate into notochord and mesenchyme autonomously as partial embryos (Kim and Nishida, 1999, Nakatani and Nishida, 1994). Gene expression profiles for these four types of embryo were analyzed by RNA sequencing (RNA-seq) (Figure 6A). There were 929 genes with significantly different expression levels between notochord and mesenchyme partial embryos (NOIseq p value adjusted for multiple testing <0.001). Among them, the expression levels of 280 genes were higher in notochord than in mesenchyme (notochord partial embryo [N-PE]-enriched genes), and those of 649 genes were higher in mesenchyme (mesenchyme partial embryo [M-PE]-enriched genes). We also compared the gene expression profiles between aDnze and adnZE embryos and similarly identified aDnze-enriched genes and adnZE-enriched genes. Among 280 notochord-enriched genes, 71 genes were commonly found in aDnze-enriched genes, whereas only 1 gene was commonly found in adnZE-enriched genes. On the other hand, among 649 mesenchyme-enriched genes, 17 genes were found in aDnze-enriched genes and 163 genes were found in adnZE-enriched genes (Figure 6B). Namely, sets of genes expressed in notochord and mesenchyme partial embryos are more similar to those in aDnze and adnZE embryos, respectively.

Figure 6C indicates that the 71 genes commonly enriched in aDnze and notochord partial embryos show quantitatively similar expression levels, as do the 163 genes commonly enriched in adnZE and mesenchyme partial embryos. Indeed, the correlation coefficients were 0.652 and 0.958 upon excluding one outlier in the notochord genes (see Discussion). Thus, the expression levels of the above-mentioned specific genes were also highly reproduced in adnZE embryos and moderately in aDnze embryos.

## Discussion

We developed a method to control nonlinear dynamic systems based on FVS, which are identified from the structure of networks. We confirmed that the dynamics of the GRN for fate specification in early *Ciona* embryo is controllable by manipulating the activities of FVS factors. The expression patterns that represent six of seven cell states observed in the embryo were actually induced in a deterministic manner. The results are consistent with the expected dynamic behavior of the multipotency of the system.

We performed our experiments to examine all possible manipulations in a binary control. The obtained marker gene expression patterns strongly suggest that six tissues were differentiated under at least one condition of the binary manipulations. This may indicate that qualitative regulation but not quantitative regulation is sufficient for fate specification of these six tissues in ascidian embryos. The RNA-seq experiments showed that adnZE embryos and mesenchyme partial embryos express specific genes at quantitatively similar levels. In contrast, although aDnze embryos and notochord partial embryos commonly expressed 71 genes specifically, their expression levels were not so well reproduced. This might be explained by the difference in conditions between natural dynamics and the artificial fixation of FVSs. Under the continuous fixation of an FVS, the expression of some genes may differ from natural conditions. Indeed, the expression level of *Brachyury*, which is a key regulatory gene for notochord differentiation (Takahashi et al., 1999) and an outlier shown in Figure 6C, was markedly higher in aDnze (reads per kilobase of transcript per million mapped reads [RPKM] = 11,897) than in notochord partial embryos (RPKM = 898).

We could not induce marker gene expression corresponding to muscle in any of the conditions that we examined. Quantitative manipulation of the FVSs may be required to induce the expression of muscle markers. Another possibility is that the GRN that we used in this study did not include factors (nodes or edges) that are responsible for the specification of muscle fate. Indeed, a previous study (Nishida and Sawada, 2001) showed that a localized maternal factor plays an important role in the specification of muscle fate. If such factors take more dominant roles than the GRN, controlling GRN alone would not be sufficient to induce muscle fate.

Differentiated tissues are generally thought to be at steady states of dynamics of gene activities, and they may be established at the tailbud stage in *Ciona*. However, the GRN analyzed in this study includes genes that are not expressed in such a late stage. Although we analyzed possible steady states of the GRN up to the late gastrula stage by fixing activities of the FVS factors, it might be difficult to observe these steady states in the actual development of *Ciona*. Indeed, expression of the FVS factors except *Foxa.a* is transient in *Ciona* embryos. However, the results of our analysis imply that artificially induced steady states of the GRN are sufficient to specify cell fates at a later stage. One possible explanation for this is as follows. In normal development, the dynamics of gene activities may fall into a steady state of the GRN by the late gastrula stage and thereafter genes downstream of the GRN may suppress the expression of the FVS factors. If this is the case, steady states for fate decision, which possibly exist in the GRN, are transient and therefore become undetectable by integrating regulation at a later stage. Our result suggests that it is practical to decompose a GRN into subnetworks and to study steady states of the subnetworks to understand cell specification processes.

In normal embryos, the GRN governs specific gene expression temporally and spatially. The important function of the GRN for specification of cell fates may be to create specific expression patterns of the FVS genes, which activate cell-type-specific downstream pathways, because a specific combination of the activities of the FVS factors determined a specific cell fate (Figure 4). Gene expression patterns of *Foxa.a*, *Foxd*, *Neurog*, and *Zic-r.b* and temporal and spatial patterns of the activity of the Erk pathway are mostly consistent with the above-mentioned speculation (Haupaix et al., 2013, Hudson et al., 2003, Imai et al., 2002a, Imai et al., 2002b, Imai et al., 2004, Ohta and Satou, 2013, Picco et al., 2007, Shi and Levine, 2008, Shimauchi et al., 2001). The epidermal markers are expressed in adnze embryos. In normal embryos, *Foxd*, *Neurog*, and *Zic-r.b* are not expressed in the epidermal lineage, and *Foxa.a* is expressed transiently only in early embryos. In addition, the Erk pathway is not turned on in this lineage before gastrulation. Mesenchyme markers were expressed in adnZE embryos. In the mesenchyme lineage of normal embryos, *Zic-r.b* is strongly expressed and the Erk pathway is activated, whereas *Foxa.a* and *Foxd* are expressed transiently only in early embryos. The notochord maker was expressed in aDnze embryos. In the notochord lineage, *Foxd, Foxa.a*, and *Zic-r.b* are expressed and the Erk pathway is activated. Although this pattern does not fully support the aforementioned hypothesis, expression patterns of Foxd, Foxa.a, and Zic-r.b proteins are not known; if Foxd is not degraded for a long time, it is possible that conditions that lead to notochord differentiation appear in the notochord lineage of normal embryos.

The structural theory provides strong predictions directly from the structure of the network without assuming other quantitative details of dynamics. Although another theory has been proposed that gives criteria to choose driver nodes in linear systems structurally (Lin, 1974, Liu et al., 2011), linkage logic is the first theory to determine key nodes for controlling nonlinear systems only from the structure of networks. Theoretically, the strategy is applicable to any nonlinear dynamic system, which includes networks other than GRNs (Fiedler et al., 2013, Mochizuki et al., 2013, Zanudo et al., 2017), and is particularly useful for controlling or engineering complex biological networks.

## Methods

All methods can be found in the accompanying Transparent Methods supplemental file.

## Supplemental Information

Document S1. Transparent Methods, Figures S1–S8, and Tables S1 and S3–S5

Table S2. Regulatory Interactions in the Developmental Gene Regulatory Network in Ciona Embryos Up to the Late Gastrula Stage, Related to Figure 2 and Transparent Methods
