# Supplementary material for: Controlling Cell Fate Specification System by Key Genes Determined from Network Structure
Source: iScience. 2018 Jun 7;4:281–93. doi: 10.1016/j.isci.2018.05.004 (PMC6147236; doi:10.1016/j.isci.2018.05.004)
Supplement: Document S1. Transparent Methods, Figures S1–S8, and Tables S1 and S3–S5 [file mmc1.pdf]

ISCI, Volume *4*

Supplemental Information

Controlling Cell Fate Specification System by Key Genes Determined from Network Structure

Kenji Kobayashi, Kazuki Maeda, Miki Tokuoka, Atsushi Mochizuki, and Yutaka Satou

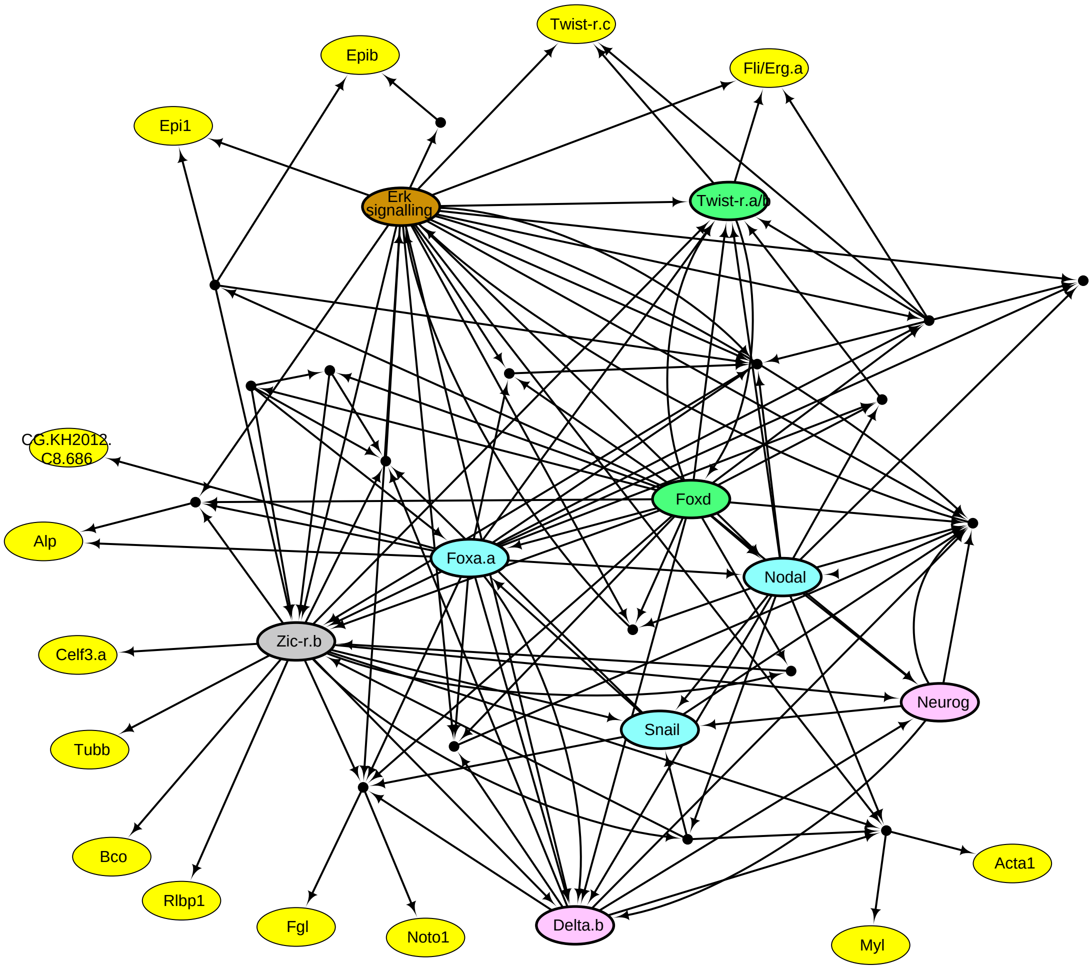

Figure S1. A simplified version of the gene regulatory network for cell specification in *Ciona*. Related to Figure 2. Only factors that are involved in directed cycles or that are directly involved in regulation of markers are shown. The FVS factors are shown by ovals colored by light blue, green, pink, grey and orange. Nodes filled in yellow are the marker genes, for which we performed observations of activities. The remaining factors are shown by black dots.

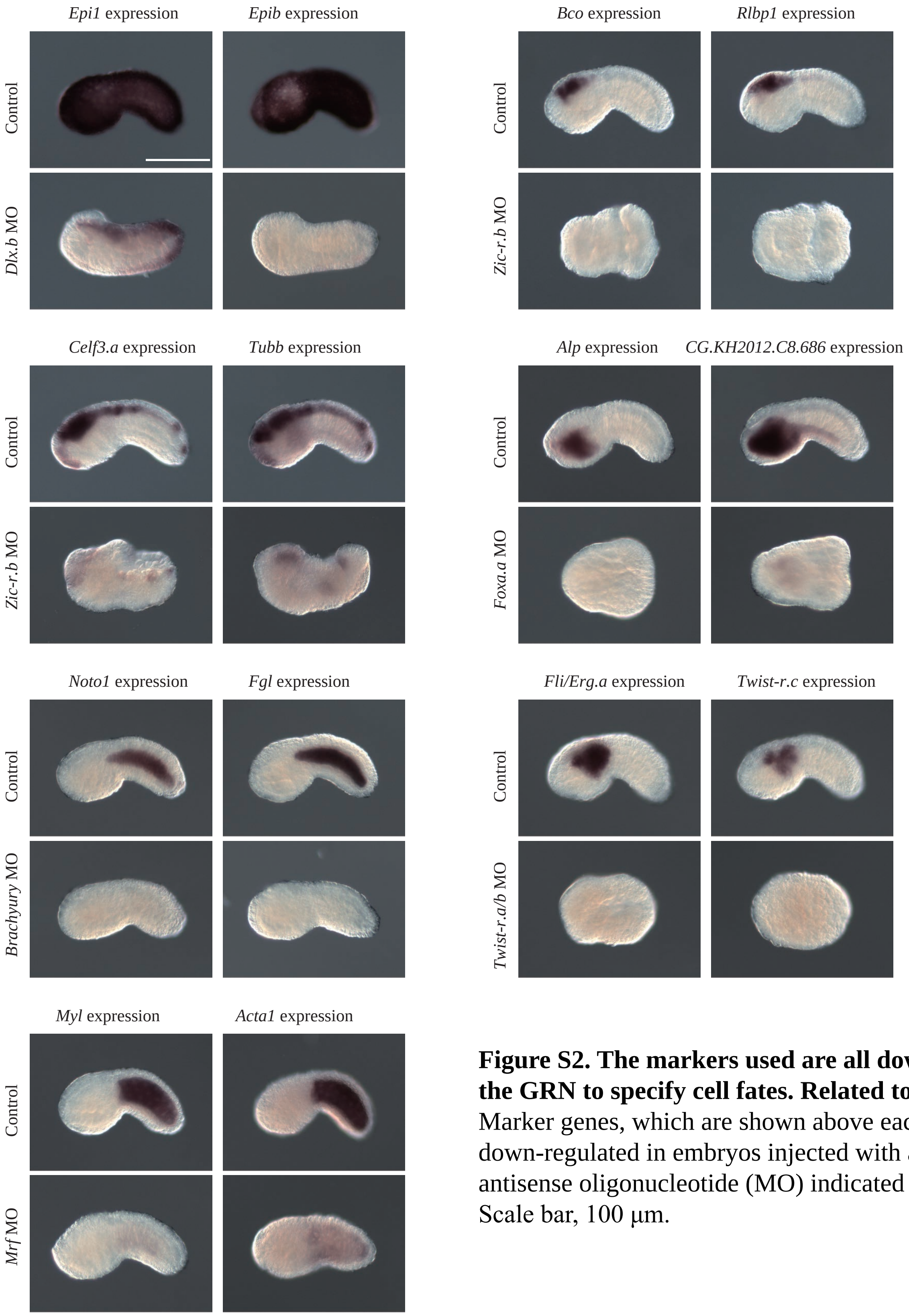

Figure S2. The markers used are all downstream of the GRN to specify cell fates. Related to Figure 2. Marker genes, which are shown above each panel, are down-regulated in embryos injected with a morpholino antisense oligonucleotide (MO) indicated on the left. Scale bar, 100 μm.

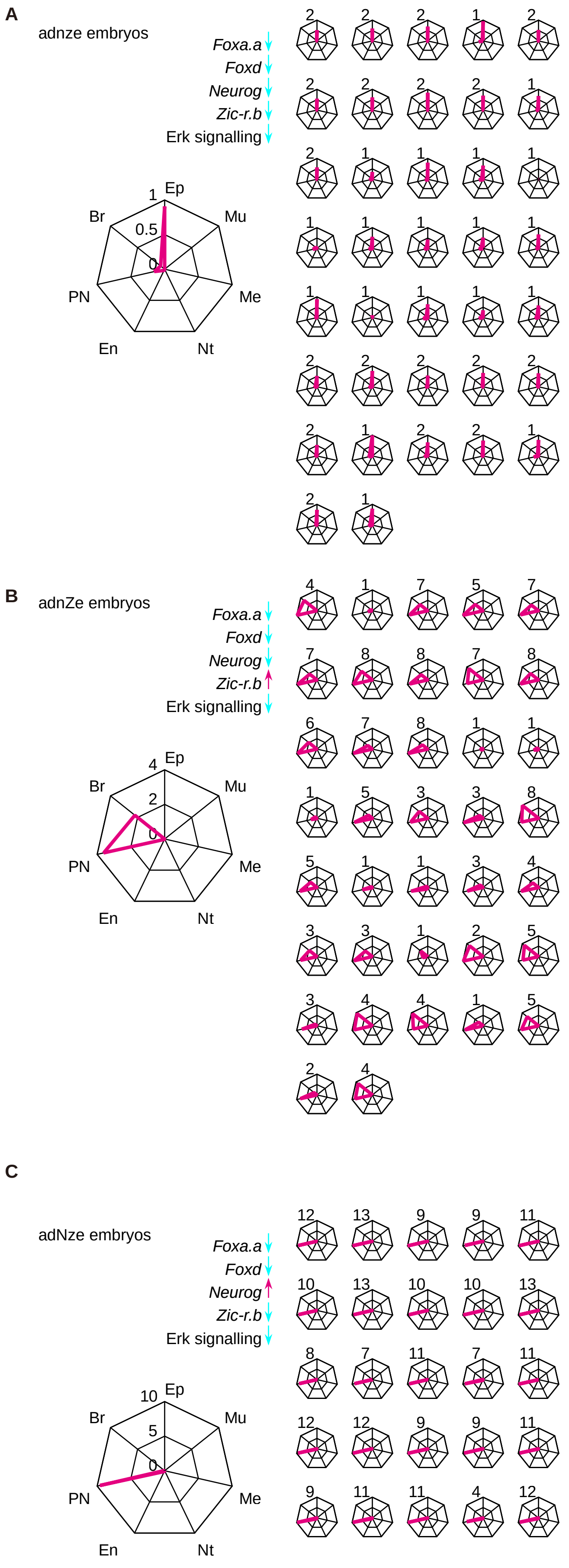

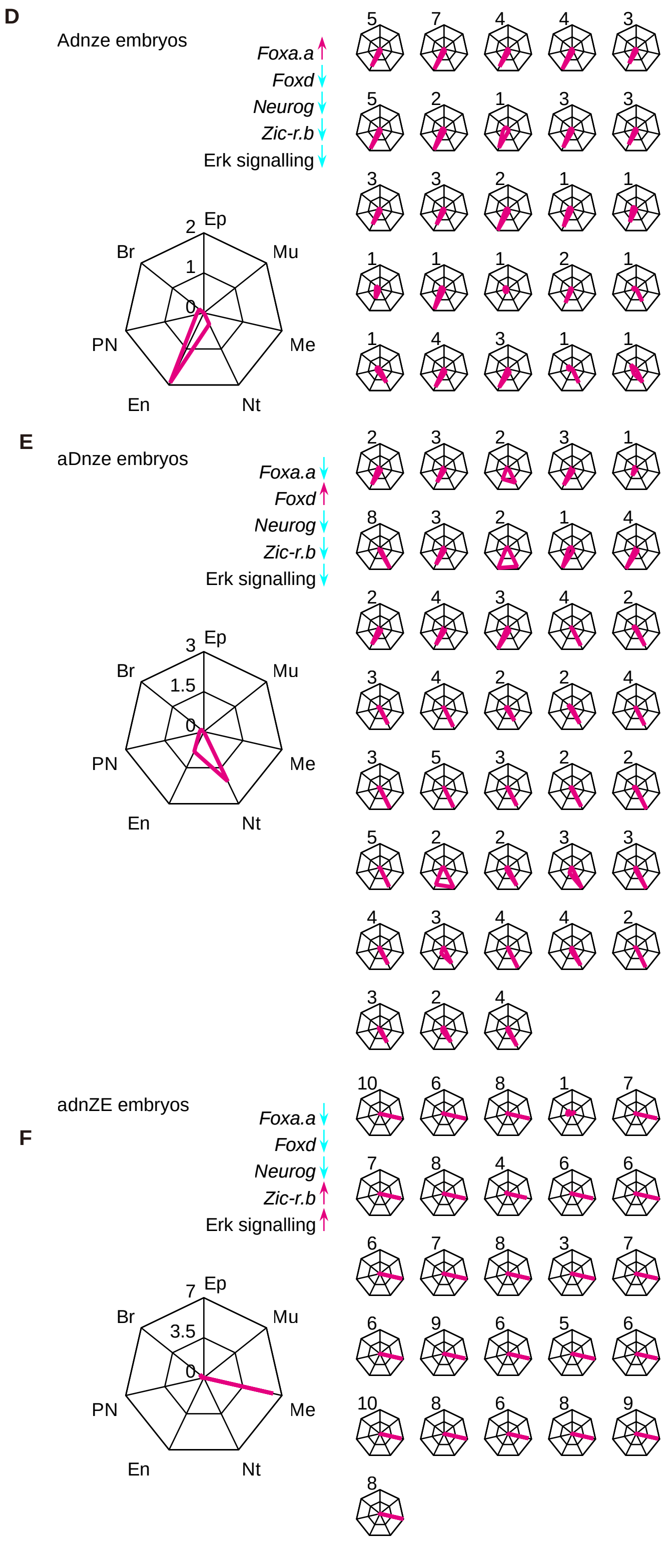

Figure S3. Marker gene expression in adnze, adnZe, adNze, Adnze, aDnze and adnZE embryos. Related to Figure 4. The large graphs on the left of each panel show the average expression levels of tissue markers, as determined by RT-qPCR. Small graphs on the right of each panel show expression levels in individual embryos. Expression levels of marker genes are shown relative to the average expression levels in normal 9.5 hr (tailbud-stage) embryos.

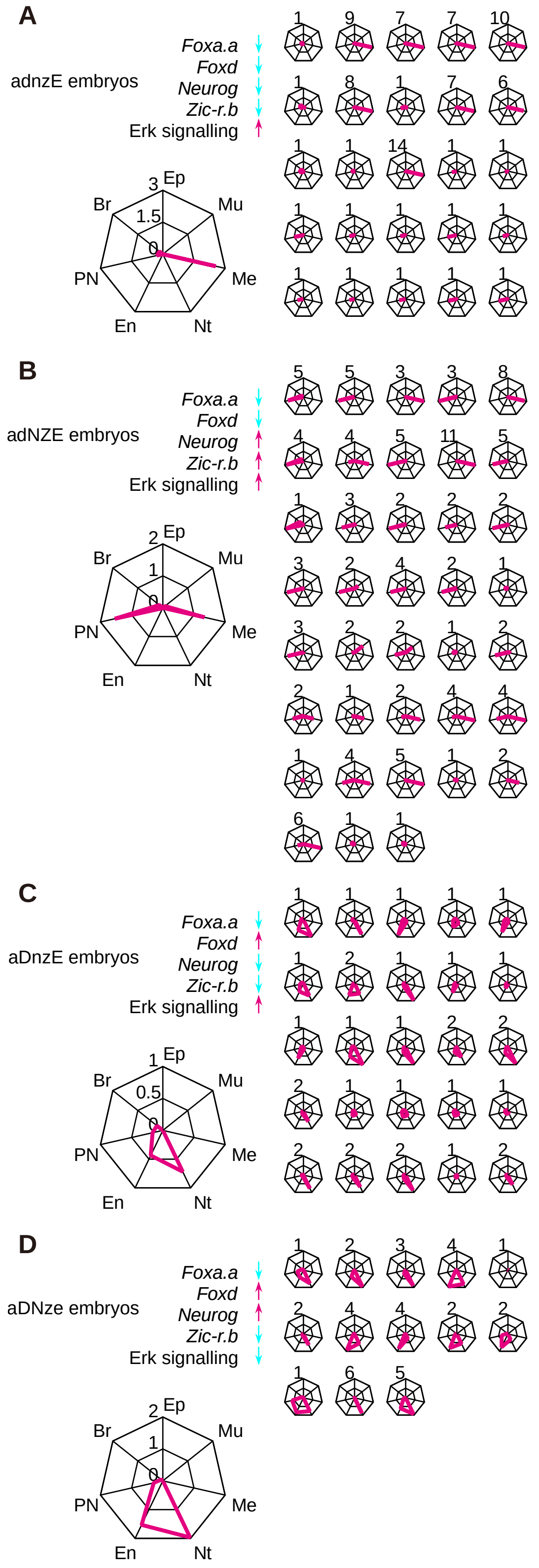

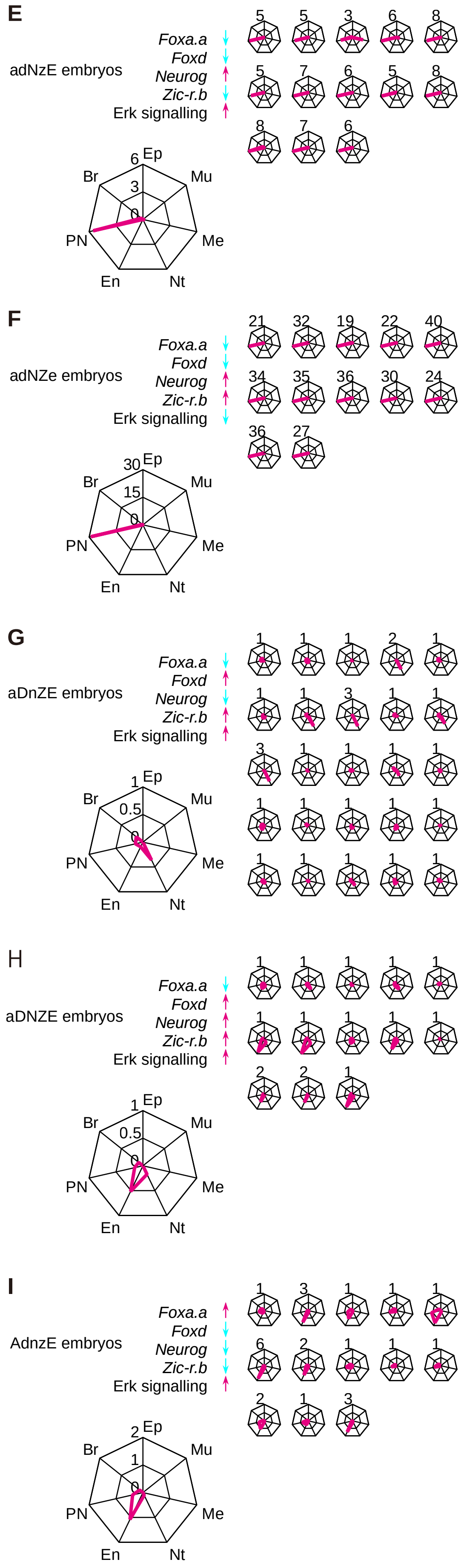

Figure S4. Marker gene expression in adnzE, adNZE, aDnzE, aDNze, adNzE, adNZe, aDnZE, aDNZE and AdnzE embryos. Related to Figure 4. The large graphs on the left of each panel show the average expression levels of tissue markers, as determined by RT-qPCR. Small graphs on the right of each panel show the expression levels in individual embryos. Expression levels of marker genes are shown relative to the average expression levels in normal 9.5 hr (tailbud-stage) embryos.

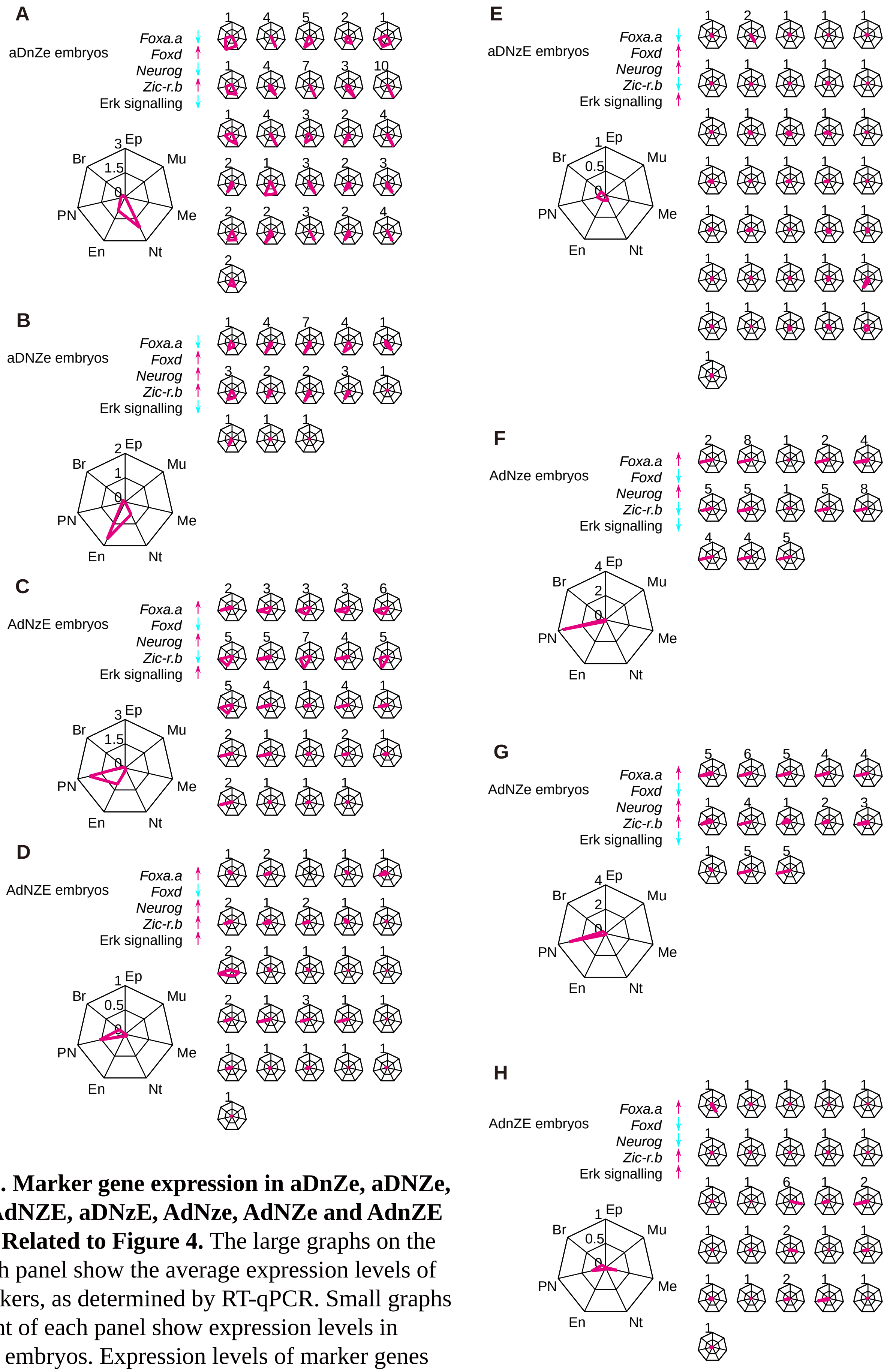

Figure S5. Marker gene expression in aDnZe, aDNZe, AdNzE, AdNZE, aDNzE, AdNze, AdNZe and AdnZE embryos. Related to Figure 4. The large graphs on the left of each panel show the average expression levels of tissue markers, as determined by RT-qPCR. Small graphs on the right of each panel show expression levels in individual embryos. Expression levels of marker genes are shown relative to the average expression levels in normal 9.5 hr (tailbud-stage) embryos.

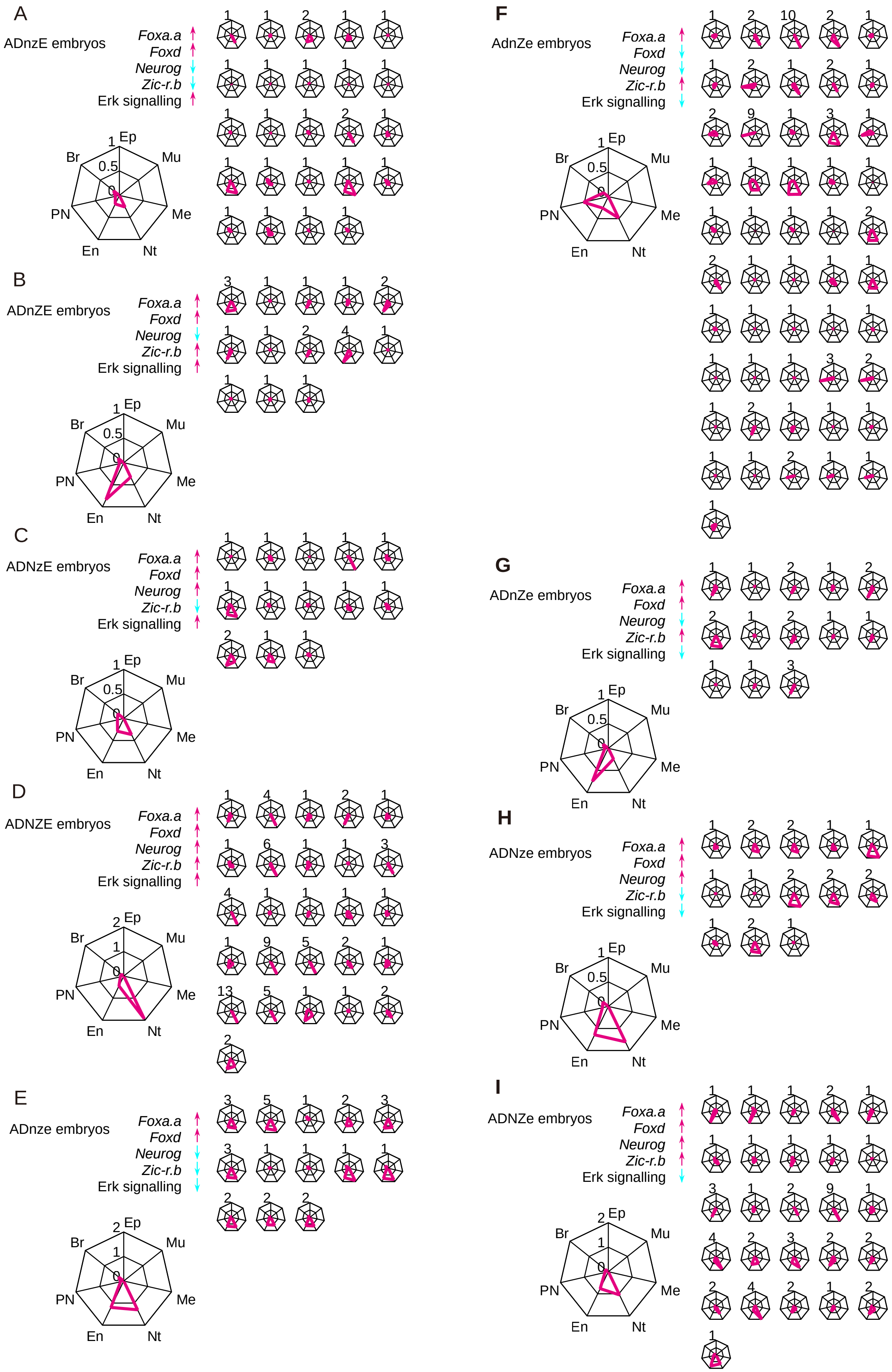

Figure S6. Marker gene expression in ADnzE, ADnZE, ADNzE, ADNZE, ADnze, AdnZe, ADnZe, ADNze and ADNZe embryos. Related to Figure 4. The large graphs on the left of each panel show the average expression levels of tissue markers, as determined by RT-qPCR. Small graphs on the right of each panel show expression levels in individual embryos. Expression levels of marker genes are shown relative to the average expression levels in normal 9.5 hr (tailbud-stage) embryos.

A

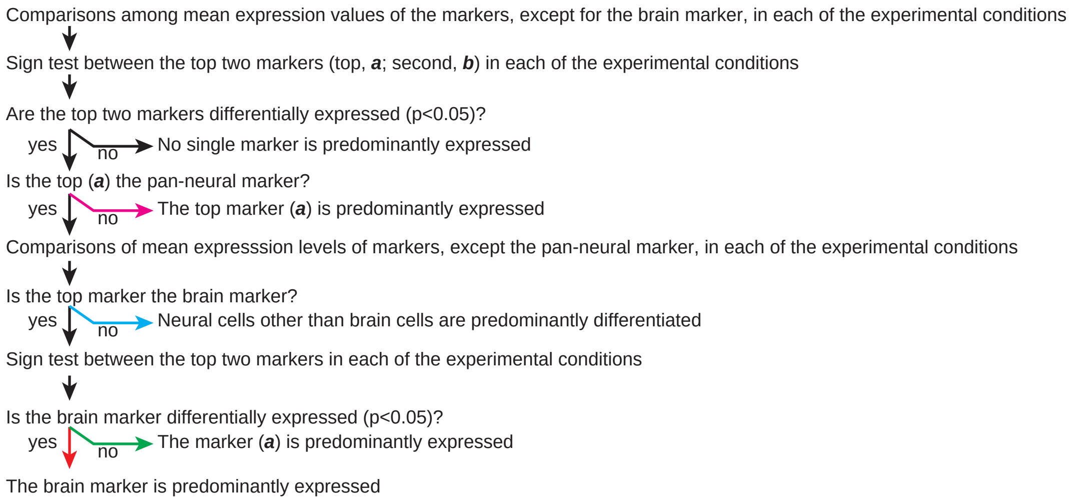

B

Experimental condition	Comparison among markers, excluding *Bco*: Top marker (***a***)	Second marker (***b***)	Sign test P-value	Comparison among markers, excluding *Celf3.a*: Top marker	Second marker	Sign test P-value	Result
adnzE	*Fli/Erg.a*	*Celf3.a*	6.90E-01				No predominantly expressed gene
adnze	*Epi1*	*Celf3.a*	1.08E-09				Epidermis
adnZE	*Fli/Erg.a*	*Celf3.a*	8.05E-07				Mesenchyme
adnZe	*Celf3.a*	*Alp*	1.46E-11	*Bco*	*Alp*	5.53E-10	Brain
adNzE	*Celf3.a*	*Fli/Erg.a*	2.44E-04	*Fli/Erg.a*	*Bco*		Pan-neural
adNze	*Celf3.a*	*Alp*	5.96E-08	*Alp*	*Bco*		Pan-neural
adNZE	*Celf3.a*	*Fli/Erg.a*	3.36E-02	*Fli/Erg.a*	*Bco*		Pan-neural
adNZe	*Celf3.a*	*Alp*	4.88E-04	*Bco*	*Alp*	4.88E-04	Brain
aDnzE	*Noto1*	*Alp*	1.00E+00				No predominantly expressed gene
aDnze	*Noto1*	*Alp*	1.39E-02				Notochord
aDnZE	*Noto1*	*Celf3.a*	1.00E+00				No predominantly expressed gene
aDnZe	*Noto1*	*Alp*	5.57E-01				No predominantly expressed gene
aDNzE	*Celf3.a*	*Alp*	1.67E-02	*Bco*	*Alp*	6.52E-02	Pan-neural*
aDNze	*Noto1*	*Alp*	1.00E+00				No predominantly expressed gene
aDNZE	*Alp*	*Noto1*	9.23E-02				No predominantly expressed gene
aDNZe	*Alp*	*Noto1*	3.42E-03				Endoderm
AdnzE	*Alp*	*Celf3.a*	1.00E+00				No predominantly expressed gene
Adnze	*Alp*	*Noto1*	9.11E-04				Endoderm
AdnZE	*Celf3.a*	*Fli/Erg.a*	3.61E-08	*Fli/Erg.a*	*Bco*		Pan-neural*
AdnZe	*Celf3.a*	*Noto1*	1.77E-03	*Noto1*	*Alp*		Pan-neural
AdNzE	*Celf3.a*	*Alp*	3.59E-05	*Alp*	*Noto1*		Pan-neural
AdNze	*Celf3.a*	*Alp*	2.44E-04	*Alp*	*Bco*		Pan-neural
AdNZE	*Celf3.a*	*Noto1*	5.96E-08	*Bco*	*Noto1*	3.59E-05	Brain*
AdNZe	*Celf3.a*	*Noto1*	2.44E-04	*Bco*	*Noto1*	9.23E-02	Pan-neural
ADnzE	*Noto1*	*Alp*	8.15E-01				No predominantly expressed gene
ADnze	*Noto1*	*Alp*	3.86E-02				Notochord
ADnZE	*Alp*	*Noto1*	2.44E-04				Endoderm
ADnZe	*Alp*	*Noto1*	6.35E-03				Endoderm
ADNzE	*Noto1*	*Alp*	6.35E-03				Notochord*
ADNze	*Noto1*	*Alp*	3.42E-03				Notochord
ADNZE	*Noto1*	*Alp*	8.45E-01				No predominantly expressed gene
ADNZe	*Noto1*	*Alp*	1.69E-01				No predominantly expressed gene

*Expression levels of all markers were low (mean expression < 0.5)

P-values: Red, p<0.05; Black, 0.05 < p

Figure S7. Markers predominantly expressed in the 32 experimental conditions. Related to Figure 4. (A) Flowchart for definition of the predominantly expressed gene in each condition. **(B)** Results of sign tests. Colours of arrows correspond to those in (A).

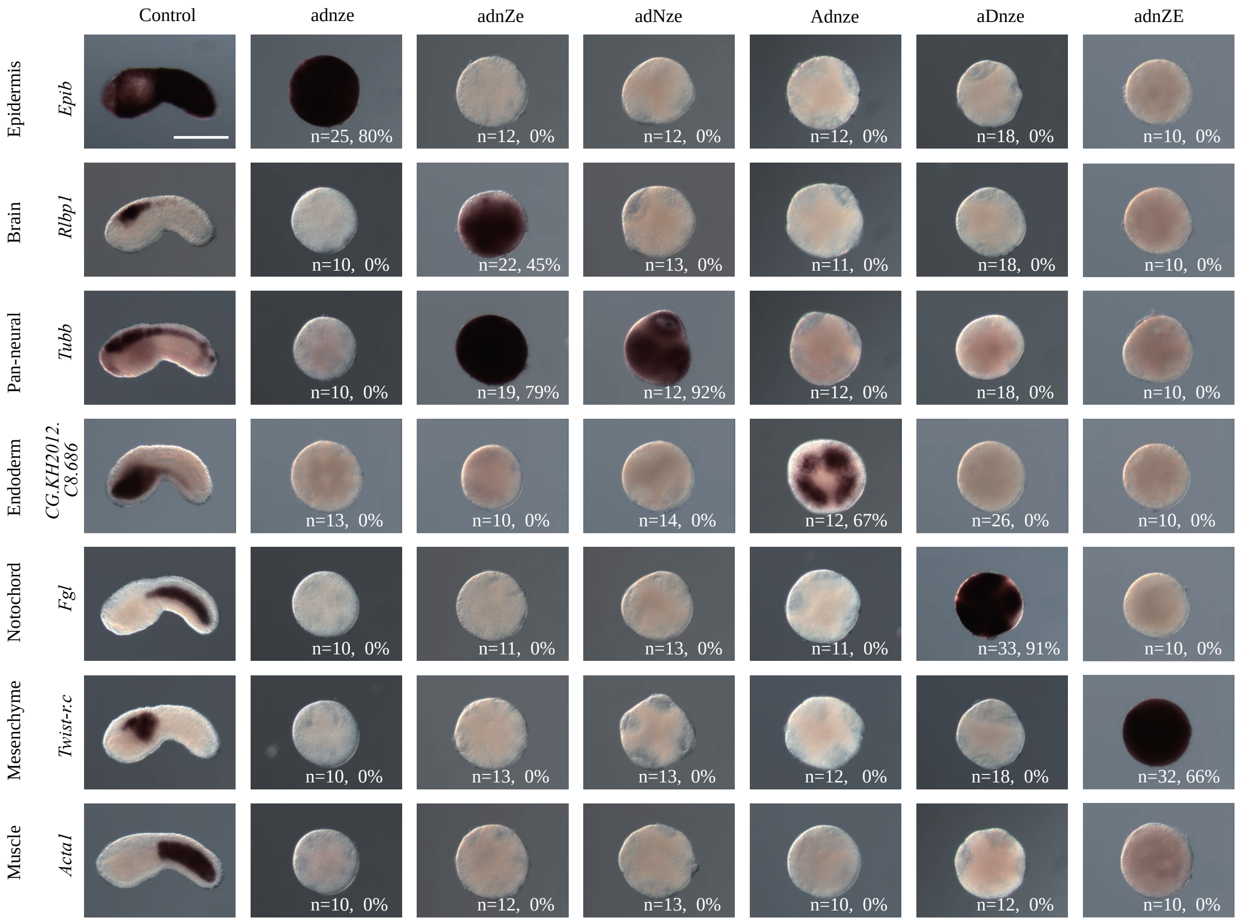

Figure S8. Expression of marker genes in adnze, adnZE, adnZe, adNze, aDnze and Adnze embryos revealed by *in situ* hybridization. Related to Figure 4. Photographs show the expression of the second set of tissue markers (shown on the left of each row). Numbers of embryos examined and percentages of embryos that expressed the specified marker are shown within each photograph. Scale bar, 100 µm.

Table S1. Mean expression levels of marker genes. Related to Table 1.

		Experimental Condition [a]								
		unperturbed [b]	adnze [b]	adnZe [b]	adNze [b]	Adnze [b]	aDnze [b]	adnZE [b]	adnz [b]	Z [b]
Expression [c]	*Epi1* (epidermis)	0.63	0.91	0.00	0.00	0.00	0.00	0.00	0.50	0.00
	Bco (brain)	2.30	0.08	2.20	0.06	0.15	0.14	0.43	0.18	2.16
	Celf3.a (pan-neural)	3.03	0.15	3.61	9.59	0.19	0.17	0.18	0.34	2.60
	Alp (endoderm)	0.01	0.02	0.03	0.13	1.94	0.83	0.01	0.04	0.03
	Noto1 (notochord)	0.15	0.01	0.01	0.00	0.31	2.07	0.00	0.01	0.01
	Fli/Erg.a (mesenchyme)	0.00	0.00	0.00	0.00	0.01	0.00	6.21	0.00	4.76
	Myl (muscle)	0.04	0.00	0.00	0.00	0.00	0.01	0.01	0.00	0.08

		Experimental Condition [a]								
		adnzE	adNZE	aDnzE	aDNze	adNzE	adNZe	aDnZE	aDNZE	AdnzE
Expression [c]	*Epi1* (epidermis)	0.00	0.00	0.00	0.00	0.02	0.01	0.00	0.00	0.00
	Bco (brain)	0.25	0.14	0.12	0.07	0.23	0.42	0.16	0.10	0.15
	Celf3.a (pan-neural)	0.25	1.54	0.16	0.29	5.55	29.05	0.14	0.15	0.38
	Alp (endoderm)	0.02	0.01	0.44	1.53	0.01	0.05	0.09	0.52	1.08
	Noto1 (notochord)	0.00	0.00	0.72	1.97	0.00	0.00	0.36	0.17	0.11
	Fli/Erg.a (mesenchyme)	2.88	1.34	0.00	0.00	0.24	0.00	0.00	0.00	0.00
	Myl (muscle)	0.00	0.09	0.01	0.01	0.01	0.01	0.01	0.01	0.00

		Experimental Condition [a]							
		aDnZe	aDNZe	AdNzE	AdNZE	aDNzE	AdNze	AdNZe	AdnZE
Expression [c]	*Epi1* (epidermis)	0.00	0.00	0.00	0.00	0.00	0.00	0.00	0.00
	Bco (brain)	0.16	0.11	0.12	0.15	0.12	0.03	0.25	0.09
	Celf3.a (pan-neural)	0.28	0.12	2.27	0.54	0.18	3.63	3.08	0.29
	Alp (endoderm)	0.95	1.69	1.10	0.05	0.12	0.15	0.11	0.04
	Noto1 (notochord)	2.11	0.57	0.12	0.05	0.12	0.01	0.12	0.03
	Fli/Erg.a (mesenchyme)	0.00	0.00	0.00	0.04	0.00	0.00	0.00	0.24
	Myl (muscle)	0.03	0.01	0.00	0.00	0.01	0.00	0.00	0.01

		Experimental Condition [a]								
		ADnzE	ADnZE	ADNzE	ADNZE	ADnze	AdnZe	ADnZe	ADNze	ADNZe
Expression [c]	*Epi1* (epidermis)	0.00	0.00	0.00	0.00	0.00	0.00	0.00	0.00	0.00
	Bco (brain)	0.15	0.14	0.15	0.12	0.27	0.12	0.13	0.14	0.15
	Celf3.a (pan-neural)	0.09	0.09	0.13	0.14	0.13	0.52	0.08	0.12	0.14
	Alp (endoderm)	0.19	0.83	0.29	0.45	1.20	0.26	0.75	0.64	0.77
	Noto1 (notochord)	0.26	0.33	0.36	1.93	1.30	0.48	0.24	0.80	1.05
	Fli/Erg.a (mesenchyme)	0.00	0.00	0.00	0.00	0.00	0.00	0.00	0.00	0.00
	Myl (muscle)	0.01	0.01	0.01	0.01	0.01	0.00	0.01	0.01	0.01

[a.] Each of the experimental conditions is represented by a five-letter code in which up- and down-regulation of *Foxa.a*, *Foxd*, *Neurog*, *Zic-r.b*, and Erk signalling are represented by A/a, D/d, N/n, Z/z, and E/e, respectively.

[b.] These results are also included in Table 1.

[c.] Expression levels of marker genes are shown relative to the corresponding values in normal 9.5 hr (tailbud-stage) embryos.

Table S2. Regulatory interactions in the developmental gene regulatory network in *Ciona* embryos up to the late gastrula stage. Related to Figure 2; Transparent Methods.

Table S3. Cycles in the network of Fig. 2 determine FVS. Related to Figure 2; Transparent Methods. C_i are minimum cycles identified by sets of nodes. An FVS I is given as a set of nodes that includes at least one member of all C_i, that is, $I \cap C_i \neq \emptyset, \quad \forall i$. The red nodes indicate our choice of FVS.

Node	C_1	C_2	C_3	C_4	C_5	C_6	C_7	C_8	C_9	C_{10}	C_{11}
Fgf8/17/18	*										
Erk signalling	*	*					*	*			*
Fgf9/16/20		*									
Foxd			*								
Twist-r.a/b			*								
Tbx6.b				*							
Zic-r.b				*	*		*		*		
Wnt5					*						
Delta.b						*		*			
Neurog						*					
Efna.d							*	*			*
Foxa.a									*	*	
Snail									*	*	*
Nodal										*	*

Table S4. Gene identifiers for genes used in the present study. Related to Related to Figure 2; Transparent Methods.

Gene	Identifier (CG.KH2012)
Acta1	C1.570
Admp	C2.421
Alp	L153.31
Bco	C9.224
BHLHA15	C3.308
Bmp2/4	C4.125
Brachyury	S1404.1
Cdx	C14.408
Celf3.a	C6.128
Cers.e	C3.255
CG.KH2012.C8.686	C8.686
Chd (*Chordin*)	C6.145
Ctnnb (*β-catenin*)	C9.53
Delta.b	L50.6
Dlx.b	L57.25
Dmrt.a	S544.3
Dusp1/2/4/5	C1.1079
Ebf3	L24.10
Efna.b	C3.202
Efna.c	C3.52
Efna.d	C3.716
Elk	C8.247
Emx	L142.14
Eph.a	C1.404
Epi1	C1.188
Epib	C7.154
Fgf8/17/18	C5.5
Fgf9/16/20	C2.125

Fgl	C1.832
Fli/Erg.a	C4.539
Fos	C11.314
Foxa.a	C11.313
Foxb	C4.341
Foxc	L57.25
Foxd	C8.890/C8.396
Foxh.a	C9.717
Fzd4	C6.162
Gata.a	L20.1
Gata.b	S696.1
Gdf1/3-r	C4.547
Gsx	C2.917
Hand-r	C1.1116
Hes.a	C1.159
Hes.b	C3.312
Hhex	L171.10
Id	C7.692/C7.157
Jun	C5.610
Lefty	C3.411
Lhx3/4	S215.4
Lmx1	C9.616
Meis	C10.174
Mesp	C3.100
Mnx1	L128.12
Mrf	C14.307
Msx	C2.957
Myl	C1.1186/C1.20
Myt1	C1.274
Neurog	C6.129
Nkx2-1/4	C10.338
Nodal	C1.99
Nog (*Noggin*)	C12.562

Noto1	L20.18
Otp	C14.377
Otx	C4.84
Pax3/7	C10.150
Pax6	C9.68
Pem1	C1.755
Pou4	C2.42
Prdm1-r.a	C12.493
Prdm1-r.b	C12.105
Rlbp1	C11.439
Sfrp1/5	L171.5
Six3/6	C10.367
Smyd1	S423.6
Snail	C3.751
Sox1/2/3	C1.99
Sox4/11/12	C7.523
Tbx2/3	L96.87
Tbx6.a	L8.11
Tbx6.b	S654.3
Tfap2-r.b	C7.43
Tp53.a	C1.573
Tp53.b	C3.713
Tubb	L116.85
Twist-r.a/b	C5.416/C5.554
Twist-r.c	C5.202
Wnt5	L152.45
Wnttun5	C9.257
Zf249	C4.182
Zf266	C1.777
Zic-r.a	C1.727
Zic-r.b	L59.12/L59.1/S816.1/S816.4

Table S5. Probes and primers used for quantitative PCR. Related to Related to Figure 3; Figure 4; Transparent Methods.

Gene	Fluorescent Probe (5' to 3')	Forward primer (5' to 3')	Reverse primer (5' to 3')
Epi1	(FAM)-ATCCTCGATATGAATGCGGTTTCCCC-(TAMRA)	CCAGACAATGGTGTTGGAAGAC	AACGCAGTGGAATTGAGTCACA
Bco	(VIC)-TCAGATCGATCCGGTGACCCTTGATACA-(TAMRA)	TCGCCATCACTGAAAGCAACT	GTGTTTCGCAAGATCAACCTTGT
Celf3.a	(FAM)-CTCGCCAGTAGCACGAACGCCC-(TAMRA)	GGCAAACCAACTGCAAACAA	CAACCATCAGGCCCTTCTTTT
Alp	(FAM)-AATCCTATTTTCGGCGCCGCTCC-(TAMRA)	CGGATCACAGCCATGTTTTTAC	CGACGAGCTTTGGATTATTAACGT
Noto1	(VIC)-CGTTCATGTACGGGTTTCTTGCAACCA-(TAMRA)	GGCTTGCCTGCGAATGG	GAGCACACGACTGCATCGTAA
Fli/Erg.a	(FAM)-ACGAGAAGGCGACCACCAATACACGA-(TAMRA)	TCCTACTACAGGGCAGGAAGCT	ACCCAAAGTATGCAACGTGTTTT
Myl	(VIC)-CGAGCCATTAACCTTAACCCAACCATTGAA-(TAMRA)	TGGATTCGATCAAGTAGGAGATGTT	CAATTTTTTGGCAGCCATATCTT

Transparent Methods

Linkage logic theory (LLT)

Formulation

Consider a directed graph $\Gamma = (V, E)$ consisting of a node set V and edge set E, and dynamics on the graph $\dot{\boldsymbol{x}} = \boldsymbol{F}(\boldsymbol{x})$ $(\boldsymbol{x}, \boldsymbol{F} \in \mathbb{R}^{|V|})$ (Fiedler et al., 2013; Mochizuki, 2008; Mochizuki et al., 2013). We assume (i) continuous differentiability of F_n, that is, $F_n \in C^1$, and (ii) dissipativity, that is, for any initial condition $x(0)$ and for a finite time $t \geq 0$, the dynamical state $x(t)$ is bounded by a positive constant C: $|x_n(t)| \leq C$. Suppose that the dynamics of activity x_n of biomolecule $n \in V$ is written in the form:

$$\begin{aligned} \dot{x}_n &= F_n(\boldsymbol{x}) \\ &= F_n\left(x_n, \boldsymbol{x}_{I_n}\right) \end{aligned} \tag{1}$$

with the third assumption (iii) decay condition:

$$\partial_1 F_n\left(x_n, \boldsymbol{x}_{I_n}\right) < 0. \tag{2}$$

In the expression, the bold face notation $\boldsymbol{x}_I$ with subset $I \subseteq V$ denotes the vector of components x_i with $i \in I$. We explicitly specify self-regulation ($n \in I_n$) and self-loop

on the graph Γ, if and only if $\partial F_n/\partial x_n$ is ‘not always negative’. Note that we omit the self-loop from I_n and Γ, if the self-regulatory influence from n to n is negative (i.e. self-repression or decay) and representable by the decay condition.

Note that the decay condition $\partial_1 F_n < 0$ does not always imply $\partial F_n/\partial x_n < 0$. If total partial derivative $\partial F_n/\partial x_n$ is not negative, we can redefine $\tilde{F}_n$ by including a self-loop via $\tilde{I}_n \equiv I_n \cup \{n\}$ as:

$$\tilde{F}_n\left(x_n, \boldsymbol{x}_{\tilde{I}_n}\right) \equiv F_n\left(\boldsymbol{x}_{I_n}\right) + x_n - x_n.$$

Therefore, $\tilde{F}_n$ instead of F_n with $\tilde{I}_n$ instead of I_n always satisfies the decay condition even if F_n itself does not. Thus, the decay condition does not limit the use of the formula of an ordinary differential equation.

Theorem and proof

Under formulations (1) and (2), we proved that a set of key nodes for dynamics is determined from the topology of the network (Fiedler et al., 2013; Mochizuki, 2008; Mochizuki et al., 2013).

Definition 1: In a directional graph $\Gamma = (V, E)$, a subset $I \subseteq V$ of nodes is called a feedback vertex set (FVS), if and only if a removal of the set $\Gamma \setminus I$ leaves a graph without directed cycles.

Definition 2: In a dynamic system, a subset $J \subseteq V$ of variables is called a set of determining nodes, if and only if two solutions satisfy $\tilde{\boldsymbol{x}}(t) - \boldsymbol{x}(t) \to 0 \ (t \to +\infty)$, whenever $\tilde{x}_n(t) - x_n(t) \to 0 \ (t \to +\infty)$ for all components $n \in J \subseteq V$(Fiedler et al., 2013; Foias and Temam, 1984).

We proved that these two different concepts are equivalent for the dynamics in a network (Fiedler et al., 2013; Mochizuki et al., 2013). In other words, observation of the long-term dynamics of the FVS I is sufficient to identify all possible attractors of an entire system. Similarly, controlling the dynamics of the FVS ($\boldsymbol{x}_I^*(t) - \boldsymbol{x}_I(t) \to 0$) is sufficient to drive the dynamics $\boldsymbol{x}(t)$ of a whole system to converge on one of any attractors $\boldsymbol{x}^*(t)$.

We explain the theorem and sketch the proof in the following. Details of the proof have been reported by Fiedler *et al.* (Fiedler et al., 2013).

Theorem: In the dynamics on the directed graphs (1) and (2), an FVS of the graph is a set of determining nodes regardless of the choice of nonlinear function F_n. Conversely, if a subset of vertices of the graph is a set of determining nodes regardless of the choice of F_n, it is an FVS.

Proof: First, we show the if-part (FVS $\Rightarrow$ determining nodes). The first step is the rearrangement of non-FVS. From the definition of FVS, nodes in a complement $K(= V \setminus I) = \{1, \dots, |K|\}$ of FVS can be aligned so that a regulating (upper) node has a smaller number than a regulated (lower) node. In other words, $I_k \subseteq I \cup \{1, \dots, k-1\}$ ($\forall k \in K$).

The second step is proof of the convergence of non-FVS under the convergence of FVS given. Let the difference of trajectories be $w_n(t) = \tilde{x}_n(t) - x_n(t)$. Suppose that $w_i(t) \to 0$ is given for all $i \in I$ included in FVS, we show $w_k(t) \to 0$ for all $k \in K$ in non-FVS via mathematical induction. For the dynamics of the difference of trajectories $\boldsymbol{w}(\mathrm{t})$, the following is induced by the mean value theorem.

$$
\begin{aligned}
\dot{\boldsymbol{w}}(t) &= \left[\boldsymbol{F}\big(\boldsymbol{x}(t)+\theta\boldsymbol{w}(t)\big)\right]_{\theta=0}^{1} \\
&= \int_0^1 \frac{d}{d\theta}\boldsymbol{F}\big(\boldsymbol{x}(t)+\theta\boldsymbol{w}(t)\big)d\theta \\
&= \int_0^1 \frac{\partial}{\partial\boldsymbol{x}}\boldsymbol{F}\big(\boldsymbol{x}(t)+\theta\boldsymbol{w}(t)\big)\cdot\boldsymbol{w}(t)d\theta \\
&= A(t)\boldsymbol{w}(t)\ \ ,
\end{aligned}
$$

where

$$
A(t) \coloneqq \left(\int_0^1 \frac{\partial \boldsymbol{F}}{\partial \boldsymbol{x}}\bigg|_{\boldsymbol{x}(t)+\theta\boldsymbol{w}(t)} d\theta\right).
$$

The A is a matrix given by integration of each element of the Jacobian of $\boldsymbol{F}$ at $\boldsymbol{x}(t)+\theta\boldsymbol{w}(t)$ with respect to θ. Note that K does not have any self-regulatory nodes, and takes a linear nonautonomous dynamic equation for each $k \in K$ as $\dot{w}_k(t) = -a_k(t)w_k(t) + \boldsymbol{b}_k^T(t)\cdot\boldsymbol{w}_{I_k}(t)$. From the assumption of dissipativity, the nonautonomous coefficients $a_k(t) \in \mathbb{R}$, $\boldsymbol{b}_k(t) \in \mathbb{R}^{|I_k|}$ are bounded by constants a_0, b_0 as $0 < a_0 \leq a_k(t)$, $|\boldsymbol{b}_k(t)| \leq b_0$. Assuming that $w_n(t) \to 0$ is already given $\forall n \in \{1,\cdots,k-1\}$, we show it for $n = k$. By solving the nonautonomous dynamic equation, we have

$$
\begin{aligned}
w_k(t) \le \exp\left(-\int_0^t a_k(s)ds\right)|w_k(0)| \\
+\sum_{j\in I_k}\int_0^t \exp\left(-\int_s^t a_k(\sigma)d\sigma\right)|\boldsymbol{b}_k(s)|\left|w_j(s)\right|ds \\
\le \exp(-a_0 t)|w_k(0)| + \sum_{j\in I_k}\int_0^t \exp\big(-a_0(t-s)\big)b_0\left|w_j(s)\right|ds \\
\to 0 \quad (t\to\infty)
\end{aligned}
$$

Here, the first term is shown by $\exp(-a_0 t) \to 0$, and the second term is shown by $w_j(s) \to 0 \ \ (s \to \infty) \ \ \forall j \in I_k \subseteq I \cup \{1, \dots, k-1\}$. In the case of $k = 1$, $I_k \subseteq I$, trivially $w_k(t) \to 0$ because $w_j(s) \to 0$ is given $\forall j \in I_k \subseteq I$. From the above, the first half of the theorem is proven.

Finally, the only-if-part (FVS $\Leftarrow$ determining nodes) is shown by taking the contrapositive, that is, a subset of vertices that is not an FVS is not a set of determining nodes. Suppose I' is not an FVS; in other words, $\Gamma \setminus I'$ contains directed cycles. By appropriate selection of the function of nodes, dynamics can be constructed so that I' is not a set of determining nodes. For example, all functions included in I' are taken to be simple decay $F_n\left(x_n, \boldsymbol{x}_{I_n}\right) \coloneqq -x_n$. From this, the behaviour of $\Gamma \setminus I'$ cannot be captured by I'. However, there is a cycle within $\Gamma \setminus I'$ and, by choosing these functions, it is

possible to create diversity in the solutions such as multiple stationary points. In other words, I', which is not an FVS, is not always a set of determining nodes for arbitrary functions.

Our theory of controllability has a broader meaning than switching between solutions that can be observed in natural conditions. For any given trajectory $\boldsymbol{x}_I^*(t)$ of an FVS, dynamics $x_k^*(t)$ of other nodes, which are not included in the FVS ($k \notin I$), converges to a unique trajectory for a long time, even if the given trajectory $\boldsymbol{x}_I^*(t)$ is not chosen from known natural solutions $s \in S$.

Identification of FVSs

The GRN is shown in Figure 2 and also given as a list of linkages (edges) connecting genes (nodes) in Table S2. Note that our theory does not require a distinction between positive and negative regulation, except for self-regulation. We omitted linkages of self-repression from the decay condition (see treatment of n in I_n). Multiple methods

and algorithms have been proposed to identify minimum FVS from network structures. Our algorithm is as follows.

We first identified nodes (genes) that are not regulated by others and nodes that do not regulate other nodes, and removed these nodes and connecting edges from the gene regulatory network repeatedly because removal of these nodes does not affect the identification of FVSs.

We next identified independent directed cycles in the graph by a 'depth-first search algorithm', starting from all nodes, examining paths through directed edges and recursively choosing all emanating edges at branching points. We repeated this process until it came back to the starting point (identification of a cycle) or reached a cycle that had already been identified. We chose a cycle with a smaller number of nodes if a pair of cycles exhibited an inclusion relationship. For each cycle, we identified a set of nodes $C_i = \{n_1^i, n_2^i, \ldots, n_{m_i}^i\}$ ($i = 1,2, \ldots, i_{\max}$) passed through by that cycle. The C_i with $i_{\max} = 11$ for the gene regulatory network of *Ciona intestinalis* is shown in Table S3. Finally, we identified sets (Is) of nodes in which at least one member of all C_i ($i = 1,2, \ldots, i_{\max}$) was included: $I \cap C_i \neq \emptyset$ for all i. We found that 12 Is were the smallest sets, each of which contained five

genes. These Is were the minimum FVSs. The minimum FVSs are {*Foxa.a*|*Nodal*|*Snail*, *Foxd*|*Twist-r.a/b*, *Neurog*|*Delta.b*, *Zic-r.b*, Erk signalling}, where '|' indicates an alternative choice.

The computer code for identifying minimum FVSs is available on https://github.com/kmaed/searchfvs.

Comparison with alternative method

Another study has already provided a criterion to choose driver nodes based on network information. The formulation and proof are given for a linear or linearized system, based on standard control theory (Kalman, 1963; Liu et al., 2011).

$$\dot{\boldsymbol{x}}(\mathrm{t}) = \mathrm{A}\boldsymbol{x}(\mathrm{t}) + \mathrm{B}\boldsymbol{u}(\mathrm{t})$$

Here, $\boldsymbol{x}(t) \in \mathbb{R}^N$ is the state vector, $\boldsymbol{u}(t) \in \mathbb{R}^M$ is the input vector, A is an $N \times N$ matrix, and B is an $N \times M$ matrix. The network structure is reflected in the distribution of nonzero entries in the matrix A. Kalman's controllability is defined as the full rankness of controllability matrix, namely:

$$\text{rank}[\text{B AB A}^2\text{B } \dots \text{ A}^{N-1}\text{B}] = N.$$

Lin (Lin, 1974) gave proof of the controllability of a linear system by introducing the concepts ‘cactus’ and ‘spanned by a cactus’. Later, Liu *et al.* (Liu et al., 2011) adapted the method to many examples of regulatory networks by an algorithm using different terminology, ‘maximum matching’. The minimum number of inputs or driver nodes needed to maintain full control of the network is determined by the ‘maximum matching’ in the network, that is, the maximum set of links that do not share both start and end nodes. A node is considered to be ‘matched’ if a link in the maximum matching points at it; otherwise, it is unmatched. If there are directed paths from the input to all matched nodes, then the system is controllable by driving unmatched nodes.

There are multiple differences between our theory (FVS control) and that proposed by Liu *et al*. (i) Our theory is applicable for any nonlinear dynamic system, even if the nonlinear functions are unknown. (ii) We use the same FVS for both observation and control. The equivalence of sets for observability and controllability realizes ‘observation-base control’. We achieve this control by prescribing the behaviour $\boldsymbol{x}_I$ on FVS I to their previously observed trajectory $\boldsymbol{x}_I^s$, $\boldsymbol{x}_I(t) - \boldsymbol{x}_I^s(t) \to 0$. The procedure is much closer to the

idea of ‘reprogramming’ regulatory networks in life sciences. (iii) Moreover, the aim and meaning of ‘control’ differ between the two methods. Liu *et al.* sought to steer the network state $x(t)$ from any initial state $\boldsymbol{x}_0$ to any target state $\boldsymbol{x}_T$ in a linear space. Instead, we seek to steer $\boldsymbol{x}(t)$ from any initial state $\boldsymbol{x}_0$ to any target solution $\boldsymbol{x}^*(t)$ of the original system (1), (2).

Animals, whole-mount *in situ* hybridization, and gene identifiers

Ciona intestinalis (type A; also called *Ciona robusta*) adults were obtained from the National Bio-Resource Project for *Ciona* in Japan. cDNA clones were obtained from our EST clone collection (Satou et al., 2005). Whole-mount *in situ* hybridization was performed as described previously (Satou et al., 1995). Gene identifiers according to the nomenclature rule (Satou et al., 2008; Stolfi et al., 2015) are shown in Table S4.

Gene knockdown and overexpression

All morpholino antisense oligonucleotides (MOs) (Gene Tools, LLC) used in the present study block translation. These MOs have been used previously and their specificity has

been evaluated (Imai et al., 2006). For synthetic mRNAs, coding sequences of *Foxa.a*, *Foxd*, *Neurogenin*, and *ZicL* were cloned into pBluescript RN3 (Lemaire et al., 1995), and synthetic mRNAs were transcribed using the mMESSAGE mMACHINE T3 Transcription Kit (Thermo Fisher Scientific). Each of the MOs was prepared at a concentration of 0.4 mM, and each mRNA was prepared at a concentration of 0.5 μg/μL. Mixtures of MOs and mRNAs were injected into eggs in volumes of 30 pL. Injection of a control MO against *E. coli lacZ* (5′-TACGCTTCTTCTTTGGAGCAGTCAT-3′) at a concentration of 1.6 mM or control *lacZ* mRNA at a concentration of 2 μg/μL yielded larvae with normal morphology. For the arrest of cell division, embryos were incubated in seawater containing 2.5 μg/mL cytochalasin B (Sigma). For up- and down-regulation of Erk signalling, we treated embryos with 10 ng/mL human recombinant basic FGF (Sigma, F0291) and 2 μM of the MEK inhibitor U0126 (Calbiochem). Reverse transcription followed by quantitative PCR was performed using the Cells-to-CT kit (Thermo Fisher Scientific). Each experimental embryo was placed into a single tube and reverse-transcription was performed in accordance with the manufacturer's instructions. Quantitative PCR was performed using the TaqMan method with primers and probes shown in Table S5.

RNA sequencing (RNA-seq)

For the RNA-seq experiments, notochord partial embryos, mesenchyme partial embryos, aDnze embryos, and adnZE embryos were collected. Two partial embryos were obtained from presumptive notochord (A7.3 and A7.7) and mesenchyme (B8.5 and B7.7) cells isolated using glass needles. RNA-seq experiments were performed as described previously

(Tokuhiro et al., 2017). NOISeq (Tarazona et al., 2011) was used to identify differentially expressed genes. We used adjusted *p*-values for multiple testing to identify differentially expressed genes, setting a threshold of 0.001.

Data Availability

The RNA-seq data is available under the SRA accession number, DRA006310.

Supplemental References

Fiedler, B., Mochizuki, A., Kurosawa, G., and Saito, D. (2013). Dynamics and Control at Feedback Vertex Sets. I: Informative and Determining Nodes in Regulatory Networks. J Dynam Differential Equations 25, 563-604.

Foias, C., and Temam, R. (1984). Determination of the Solutions of the Navier-Stokes Equations by a Set of Nodal Values. Math Comput 43, 117-133.

Imai, K.S., Levine, M., Satoh, N., and Satou, Y. (2006). Regulatory blueprint for a chordate embryo. Science 312, 1183-1187.

Kalman, R.E. (1963). Mathmatical Description of Linear Dynamical Systems. J SIAM Control Ser A 5, 152-192.

Lemaire, P., Garrett, N., and Gurdon, J.B. (1995). Expression cloning of Siamois, a Xenopus homeobox gene expressed in dorsal-vegetal cells of blastulae and able to induce a complete secondary axis. Cell 81, 85-94.

Lin, C.T. (1974). Structural Controllability. Ieee T Automat Contr Ac19, 201-208.

Liu, Y.Y., Slotine, J.J., and Barabasi, A.L. (2011). Controllability of complex networks. Nature 473, 167-173.

Mochizuki, A. (2008). Structure of regulatory networks and diversity of gene expression patterns. Journal of theoretical biology 250, 307-321.

Mochizuki, A., Fiedler, B., Kurosawa, G., and Saito, D. (2013). Dynamics and control at feedback vertex sets. II: a faithful monitor to determine the diversity of molecular activities in regulatory networks. Journal of theoretical biology 335, 130-146.

Satou, Y., Kawashima, T., Shoguchi, E., Nakayama, A., and Satoh, N. (2005). An integrated database of the ascidian, Ciona intestinalis: Towards functional genomics. Zool Sci 22, 837-843.

Satou, Y., Kusakabe, T., Araki, S., and Satoh, N. (1995). Timing of Initiation of Muscle-Specific Gene-Expression in the Ascidian Embryo Precedes That of Developmental Fate Restriction in Lineage Cells. Dev Growth Differ 37, 319-327.

Satou, Y., Mineta, K., Ogasawara, M., Sasakura, Y., Shoguchi, E., Ueno, K., Yamada, L., Matsumoto, J., Wasserscheid, J., Dewar, K., et al. (2008). Improved genome assembly and evidence-based global gene model set for the chordate Ciona intestinalis: new insight into intron and operon populations. Genome Biol 9, R152.

Stolfi, A., Sasakura, Y., Chalopin, D., Satou, Y., Christiaen, L., Dantec, C., Endo, T., Naville, M., Nishida, H., Swalla, B.J., et al. (2015). Guidelines for the nomenclature of genetic elements in tunicate genomes. Genesis 53, 1-14.

Tarazona, S., Garcia-Alcalde, F., Dopazo, J., Ferrer, A., and Conesa, A. (2011). Differential expression in RNA-seq: A matter of depth. Genome Res 21, 2213-2223.

Tokuhiro, S., Tokuoka, M., Kobayashi, K., Kubo, A., Oda-Ishii, I., and Satou, Y. (2017). Differential gene expression along the animal-vegetal axis in the ascidian embryo is maintained by a dual functional protein Foxd. PLoS genetics 13, e1006741.
